# Loop diuretics in adult intensive care patients with fluid overload: a systematic review of randomised clinical trials with meta-analysis and trial sequential analysis

**DOI:** 10.1186/s13613-022-01024-6

**Published:** 2022-06-13

**Authors:** Sine Wichmann, Marija Barbateskovic, Ning Liang, Theis Skovsgaard Itenov, Rasmus Ehrenfried Berthelsen, Jane Lindschou, Anders Perner, Christian Gluud, Morten Heiberg Bestle

**Affiliations:** 1 Department of Anaesthesiology, Copenhagen University Hospital − North Zealand, Dyrehavevej 29, 3400 Hillerød, Denmark; 2 Copenhagen Trial Unit, Centre for Clinical Intervention Research, Copenhagen University Hospital—Rigshospitalet, Blegdamsvej 9, 2100 Copenhagen, Denmark; 3 Institute of Basic Research in Clinical Medicine, China Academy of Chinese Medical Sciences, 16 Nanxiaojie, Dongzhimen, Beijing, 100700 China; 4 Department of Intensive Care, Copenhagen University Hospital − Rigshospitalet, Blegdamsvej 9, 2100 Copenhagen, Denmark; 5 Department of Regional Health Research, The Faculty of Health Sciences, University of Southern Denmark, Campusvej 55, 5230 Odense, Denmark; 6 Department of Clinical Medicine, University of Copenhagen, Blegdamsvej 3B, 2200 Copenhagen, Denmark

**Keywords:** Critical care, Diuretics, Fluid accumulation, Fluid overload, Furosemide, Loop diuretics, Systematic review

## Abstract

**Background:**

Fluid overload is a risk factor for organ dysfunction and death in intensive care unit (ICU) patients, but no guidelines exist for its management. We systematically reviewed benefits and harms of a single loop diuretic, the predominant treatment used for fluid overload in these patients.

**Methods:**

We conducted a systematic review with meta-analysis and Trial Sequential Analysis (TSA) of a single loop diuretic vs. other interventions reported in randomised clinical trials, adhering to our published protocol, the Cochrane Handbook, and PRISMA statement. We assessed the risks of bias with the ROB2-tool and certainty of evidence with GRADE. This study was registered in the International Prospective Register of Systematic Reviews (PROSPERO) (CRD42020184799).

**Results:**

We included 10 trials (804 participants), all at overall high risk of bias. For loop diuretics vs. placebo/no intervention, we found no difference in all-cause mortality (relative risk (RR) 0.72, 95% confidence interval (CI) 0.49–1.06; 4 trials; 359 participants; *I*^2^ = 0%; TSA-adjusted CI 0.15–3.48; very low certainty of evidence). Fewer serious adverse events were registered in the group treated with loop diuretics (RR 0.81, 95% CI 0.66–0.99; 6 trials; 476 participants; *I*^2^ = 0%; very low certainty of evidence), though contested by TSA (TSA-adjusted CI 0.55–1.20).

**Conclusions:**

The evidence is very uncertain about the effect of loop diuretics on mortality and serious adverse events in adult ICU patients with fluid overload. Loop diuretics may reduce the occurrence of these outcomes, but large randomised placebo-controlled trials at low risk of bias are needed.

**Supplementary Information:**

The online version contains supplementary material available at 10.1186/s13613-022-01024-6.

## Introduction

Intensive care patients receive substantial amounts of fluids during resuscitation, as maintenance fluid, with medicine, and nutrition. Large fluid input, capillary leak, and acute kidney injury (AKI) with accompanying oliguria often results in sodium chloride and water accumulation leading to fluid overload. Large iatrogenic sodium load is contributing to development of fluid overload. Sodium intake is mainly caused by isotonic maintenance fluid therapy and fluid creep from sodium containing fluids used as drug dissolvents [1]. The kidneys have a limited capacity to excrete sodium and adapts slowly (days) to substantial changes in sodium intake [1]. A high sodium intake will lead to subsequent water retention and contribute to fluid overload. Large volume fluid resuscitation and a positive fluid balance are associated with sepsis, severe burns, severe pancreatitis, and emergency surgery complicated with intraabdominal hypertension.

Fluid overload affects all organs and is an independent risk factor for intraabdominal hypertension [2–4] and the development of AKI [5–8]. AKI occurs in up to 57% of patients in the intensive care unit (ICU) [9]. Furthermore, fluid overload is associated with increased mortality in the general ICU population [10], including those with recent surgery [11, 12], sepsis [13–15], AKI [16–20], respiratory failure [21], and traumatic brain injury [22].

In an American study, diuretics were used in 49% of all patients admitted to the ICU. The loop diuretic furosemide was the predominant diuretic used in about 94% of diuretic-treated patients [23]. A multi-national study of ICU patients with AKI reported administration of diuretics in 61% of the patients and 98% of these patients received furosemide [24]. Only a minority of patients receive combinations of two or more types of diuretics [23–25].

No systematic reviews have assessed the benefits and harms of loop diuretics in the treatment of fluid overload in the ICU and no guidelines exist. With the present systematic review, our primary aim is to assess the existing evidence on all-cause mortality, quality of life, and serious adverse events from randomised clinical trials (RCT) on the treatment of fluid overload with loop diuretics in adult ICU patients [26].

## Methods

This systematic review was conducted according to our published protocol and statistical analysis plan [26]. The protocol was registered in the International Register of Systematic Reviews Database PROSPERO (CRD42020184799). We adhered to the methodology recommended by The Cochrane Collaboration [27] and used an eight-step procedure to assess if the threshold for statistical and clinical significance were crossed [28]. The steps include: both fixed-effect and random-effects model meta-analyses, subgroup analyses, sensitivity analyses, adjusted thresholds for significance, calculated realistic diversity-adjusted required information sizes using Trial Sequential Analysis, Bayes factor, assessed the impact of bias including publication bias, and clinical significance [28]. In addition, we assessed the certainty of evidence with Grading of Recommendations, Assessments, Developments and Evaluations (GRADE) [29] system and reported the review as recommended by Preferred Reporting Items for Systematic Reviews and Meta-Analysis (PRISMA) [30] (Additional file 1: S1).

### Eligibility criteria

RCTs assessing adult ICU patients with fluid overload treated with the following four comparisons were included: (1) Single loop diuretic compared with placebo or no intervention (standard of care or no diuretics). (2) Single loop diuretic compared with other types of diuretics. (3) Single loop diuretic compared with other pharmacological interventions. (4) Higher-dose loop diuretic compared with lower dose loop diuretic. We accepted any dose, formulation, timing, and duration of intervention [26].

### Outcomes

#### Primary outcomes

(1) All-cause mortality; (2) health-related quality of life; (3) proportion of participants with one or more serious adverse events (SAEs) according to either the definition from Good Clinical Practice Guideline of the International Conference on Harmonization (ICH-GCP) [31], the trialist’s definition of ‘serious adverse event’, or available data that clearly fulfilled the ICH-GCP definitions for a SAE.

#### Secondary outcomes

(1) Plasma concentration of creatinine; (2) proportion of participants without resolution of fluid overload; (3) number of days on mechanical ventilation; (4) length of stay in days in the ICU; (5) proportion of participants with adverse events not considered serious (AE).

#### Explorative outcomes

(1) Single SAEs; (2) single AEs; (3) plasma concentration of sodium, potassium, and chloride.

All outcomes were assessed at longest follow-up.

### Search methods for identification of trials

We searched the following databases: Cochrane Central Register of Controlled Trials (CENTRAL) in The Cochrane Library, MEDLINE (Ovid), Embase (Ovid), PubMed, Science Citation Index (Web of Science), Biosis Previews (Web of Science), Latin American Caribbean Health Sciences Literature (LILACS), China National Knowledge Infrastructure (CNKI), Wanfang Data, VIP Chinese Science Journals Database, and Sinomed. A search in Google Scholar was also performed.

Ongoing and unpublished trials were searched from databases of clinical trial registries and United States Food and Drug Administration (FDA) and European Medicines Agency (EMA) [26].

We applied no restrictions according to language, publication status, or year. The literature searches were last updated on April 13, 2021. Detailed search strategy in Additional file 1: S2.

### Trial selection and data extraction

Three authors (SW, MB, NL) independently screened titles and abstracts for eligibility in Covidence.org [32]. Selected articles were evaluated in full text for inclusion in accordance with the inclusion criteria by at least two authors. Disagreements were resolved by consensus.

Two investigators (SW, MB) independently extracted data from the included trials in a predefined data collection form. The following data were collected: (1) Trial: country, date of publication, duration, design (multi- or single-centre trial). (2) Participants: number of patients randomised, analysed, and lost to follow-up/withdrawn, type of patients, sex, age, inclusion and exclusion criteria. (3) Interventions: type of intervention, comparator, and concomitant interventions. (4) Outcomes: specified primary, secondary, and explorative outcomes. (5) Trial funding and notable conflicts of interest [26].

### Risk of bias

Two authors (SW, MB) independently assessed the risk of bias of all included trials and outcomes using The Cochrane Collaboration’s risk of bias tool, RoB2, by answering all the signalling questions in the five domains [33]. Disagreements were resolved by consensus. All outcomes were judged at overall low risk of bias if all five domains were at low risk of bias. Outcomes were judged at overall high risk of bias when some concerns or high risk of bias was judged in one or more domains [26].

We planned to assess bias across trials by inspecting funnels plot for asymmetry when 10 or more trials were included in a meta-analysis and tested by Harbord’s test [34] for dichotomous outcomes and with regression analysis [35] for continuous outcomes.

### Data synthesis

#### Association measures

Risk ratios (RR) were calculated for dichotomous outcomes with 95% confidence interval (CI) and Trial Sequential Analysis (TSA)-adjusted CI. End-scores were used for continuous outcomes and mean difference (MD) with 95% CIs, and TSA-adjusted CIs were calculated.

#### Meta-analyses

The effect measures were analysed using Review Manager 5 [36]. The intervention effect was calculated using both fixed-effect model with the Mantel–Haenszel method and random-effects model with the DerSimonian and Laird method. We drew conclusions based on the most conservative estimates of the two [26, 28]. For the primary outcomes, we calculated the Bayes factor [28].

#### Dealing with missing data

Corresponding authors of the trials were contacted and asked for clarifications regarding methods, data, or missing data. We received raw data from one trial [37]. We conducted sensitivity analyses to assess the potential impact of missing data by calculating a best–worst case scenario and a worst-best case scenario [26, 28].

#### Assessment of heterogeneity

Visual inspection of forest plots, inconsistency (*I*^2^) statistic, and diversity (*D*^2^) statistic were used to assess statistical heterogeneity [38]. Subgroup analyses were performed to explore clinical and statistical heterogeneity by Chi-squared test with a significance level at *P* < 0.1 [26].

#### Subgroup analyses

We planned to perform the following subgroup analyses [26]: (1) Trials at overall high risk of bias compared to trials at overall low risk of bias. (2) Type of ICU (medical ICU compared to surgical ICU and to mixed ICU). (3) Severity of fluid overload (up to 5% compared to 6% to 10% and to above 10%). (4) Type of patients according to ICU diagnose (mixed diagnoses compared to AKI, to decompensated heart failure, and to acute lung injury (ALI)/acute respiratory distress syndrome (ARDS)). Due to few included trials and sparse data, we were only able to conduct subgroup analyses according to ICU diagnoses, type of ICU, and severity of fluid overload.

We conducted a post hoc subgroup analysis for the comparison of loop diuretics vs. placebo/no intervention. The control groups in this comparison consisted of placebo, no diuretics, and standard of care. Some trials with placebo or no diuretics as control group reported administration of loop diuretics as escape or protocol violations. In standard of care, diuretics are expected to be allowed. To investigate if administration of loop diuretics in the control group had an impact on the result, we made a post hoc subgroup analysis comparing trials that reported administration of loop diuretics in the control group to trials not reporting administration of loop diuretics in the control group. Further details in Additional file 1: S3.

#### Trial sequential analysis

TSA is used to control the risks of random errors and to test if the meta-analysis had reached the required number of randomised patients to reject or accept the a priory stipulated intervention effect [38–48]. If accrued information size is too small compared to the required information size, the TSA-adjusted CI becomes wider than the traditional 95% CI, and the threshold for statistical significance will be further restricted. If the required information size is reached, the TSA-adjusted CI will be equal to the traditional naïve 95% CI for the tested intervention effect. We used a relative risk reduction (RRR) of 20% for dichotomous outcomes and minimal relevant difference of 0.5 of the observed standard deviation for continuous outcomes [28]. We used a familywise error rate of 5% [28], leading to an alpha of 0.025% for the three primary outcomes and 0.017% for the five secondary outcomes, and a beta of 10% resulting in a power of 90%.

#### Grading certainty of evidence

We used “The Grading of Recommendations Assessment, Development and Evaluation” (GRADE) approach to assess the certainty of the body of evidence associated with the predefined outcomes [49–51].

## Results

### Trial selection

**Fig. 1:**
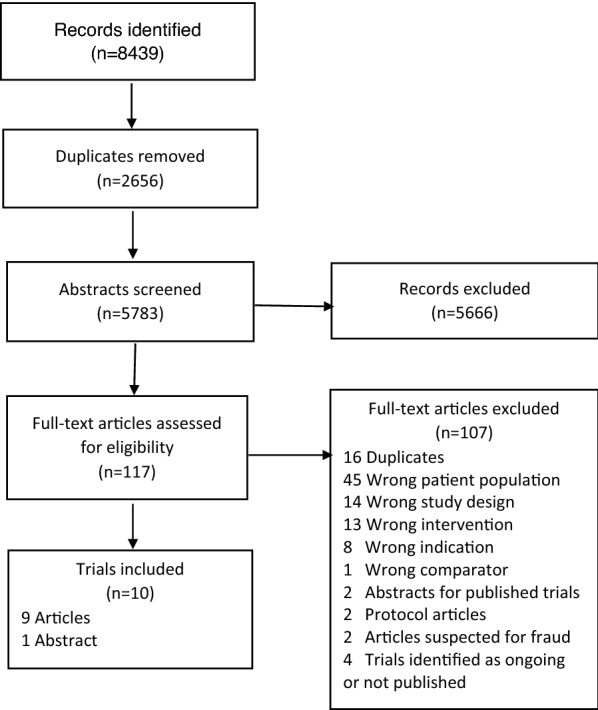
Preferred reporting items for systematic reviews and meta-analyses (PRISMA) flow chart

We identified 8338 titles and assessed 109 full text papers for eligibility (Fig. 1). We included 10 RCTs with a total of 804 participants—one text in German and nine texts in English [37, 52–60]. One trial was only published as an abstract [57]. We also identified four ongoing or unpublished trials of relevance [61–64]. No data on unpublished trials were available for this review.

### Characteristics of the included trials

We were only able to include trials investigating loop diuretics vs. placebo/no intervention (six trials), loop diuretics vs. another loop diuretic (two trials), and loop diuretics vs. another type of diuretics (two trials). All trials were small ranging from 12 to 248 participants. As experimental intervention, nine trials used furosemide and one trial used torsemide. The control group interventions consisted of: no diuretics [54, 56, 57]; placebo [52]; standard of care [37, 55]; a different loop diuretic (piretanide, ethacrynic acid) [58, 59]; or a different group of diuretics (tolvaptan, acetazolamide) [53, 60]. Albumin is the carrier for furosemide and hypoalbuminemia might result in decreased effect of the drug. None of the trials presented data on albumin levels. Further details about the trials can be found in Table 1 and Additional file 1: S4.

**Table 1:** Characteristics of included trials

Trial/year		Country	Sample size	Setting	Population	Experimental intervention	Comparator	Vasopressor treatment*	Duration of intervention	Primary outcome
	Loop diuretics vs. placebo/no intervention									
Bagshaw 2017 [52]		Canada Australia	73	Mixed ICU	AKI	Furosemide bolus of 0.4 mg/kg followed by continuous infusion with starting dose of 0.05 mg/kg/hour. Goal directed titration. Max. 0.4 mg/kg/hour	In fusion of placebo (saline)	Yes (62.6%)	Max. 7 days	Worsening of AKI
Berthelsen 2018 [37]		Denmark	23	Mixed ICU	Moderate to severe AKI and > 10% of fluid overload	40 mg of furosemide iv followed by infusion of max. 40 mg/hour. If furosemide was not efficient enough according to protocol dialysis was initiated	Standard of care	Yes (100%)	5 days	Cumulative fluid balance 5 days after randomisation
Cardoso 2013 [55]		Brazil	72	Cardiac ICU	Decompensated heart failure	120 mg of furosemide followed by titration according to effect	Standard of care	Not reported^a^	10 days	Time to being free from congestion
Cinotti 2021 [54]		France	171	Mixed ICU	Mixed ICU patients	Furosemide 1–2 times a day. Max. 250 mg	No diuretics	Exclusion criterion	Until extubation or max. 28 days	Fluid balance. It was defined as weight variation from weight on randomisation to weight on successful extubation
Hamishehkar 2017 [56]		Iran	100	Surgical ICU	AKI	40–80 mg furosemide injection followed by infusion of 1–5 mg/hour	No diuretics	Yes (22%)	7 days	AKI
Sanchez 2003 [57]		Spain	40	Not described	AKI	Torsemide (dose not described)	Control not described	Yes^b^	Max. 7 days	Creatinine and need for RRT
	Loop diuretics vs. other loop diuretics									
Han 2019 [59]		China	248	Cardiac ICU	Not described	Furosemide: 0.8 mg kg/hour	Ethacrynic acid: 0.5 mg/kg/hour	Not reported	Max. 3 days	Urine output
Wappler 1991 [58]		Germany	12	Surgical ICU	Post cardiac surgery with decompensated heart failure	Furosemide bolus of 40 mg followed by infusion of 20 mg/hour. Extra bolus of 40 mg of furosemide was allowed if the diuresis was too low	Piretanide bolus of 12 mg followed by infusion of 6 mg/hour. Extra bolus of 12 mg was allowed if the diuresis was too low	Yes (100%)	40 h	Fluid balance and electrolytes
	Loop diuretics vs. other diuretics									
Ng 2020 [60]		USA	33	Cardiac ICU	Decompensated heart failure	Infusion of furosemide 5 mg/hour. Escalation possible after 24 h to a maximum of 20 mg/hour. Metolazone was allowed if the diuresis was less than protocolised on max. furosemide	Tablet tolvaptan 30 mg once a day. Escalation possible after 24 h to maximum 60 mg/day. Metolazone was allowed if the diuresis was less than protocolised on max. tolvaptan	Exclusion criterion	Max. 4 days	Urine output 24 h post randomisation
Brown 2019 [53]		Australia	25	Mixed ICU	Mixed ICU patients	40 mg furosemide injection	500 mg acetazolamide injection	Not reported	6 h	Urine output

*ICU* intensive care unit; *AKI* acute kidney injury, *RRT* renal replacement therapy

*Vasopressor treatment at baseline

^a^No vasopressor but 69.3% received dobutamine

^b^Unclear how many patients received vasopressor

Four trials primarily presented data as medians with interquartile range (IQR) because of skewed data [37, 53, 54, 60]. This format of data is not suitable for meta-analysis. The trials were small so it was not appropriate to apply the Wan method to approximate standard deviations [65]. We, therefore, described the data narratively.

### Risk of bias

All outcomes in all trials were assessed to be at overall high risk of bias (Additional file 1: S5, S6a, S7a, S8a). With less than ten included trials in the meta-analyses, funnels plot and statistical analyses for asymmetry were not conducted. The trials were generally small. We could not assess publication bias.

### Results for loop diuretics vs. placebo/no intervention

Six trials compared a loop diuretic (five trials with furosemide and one trial with torsemide) vs. placebo [52], no diuretics [54, 56, 57], or standard of care [37, 55].

#### All-cause mortality

**Fig. 2:**
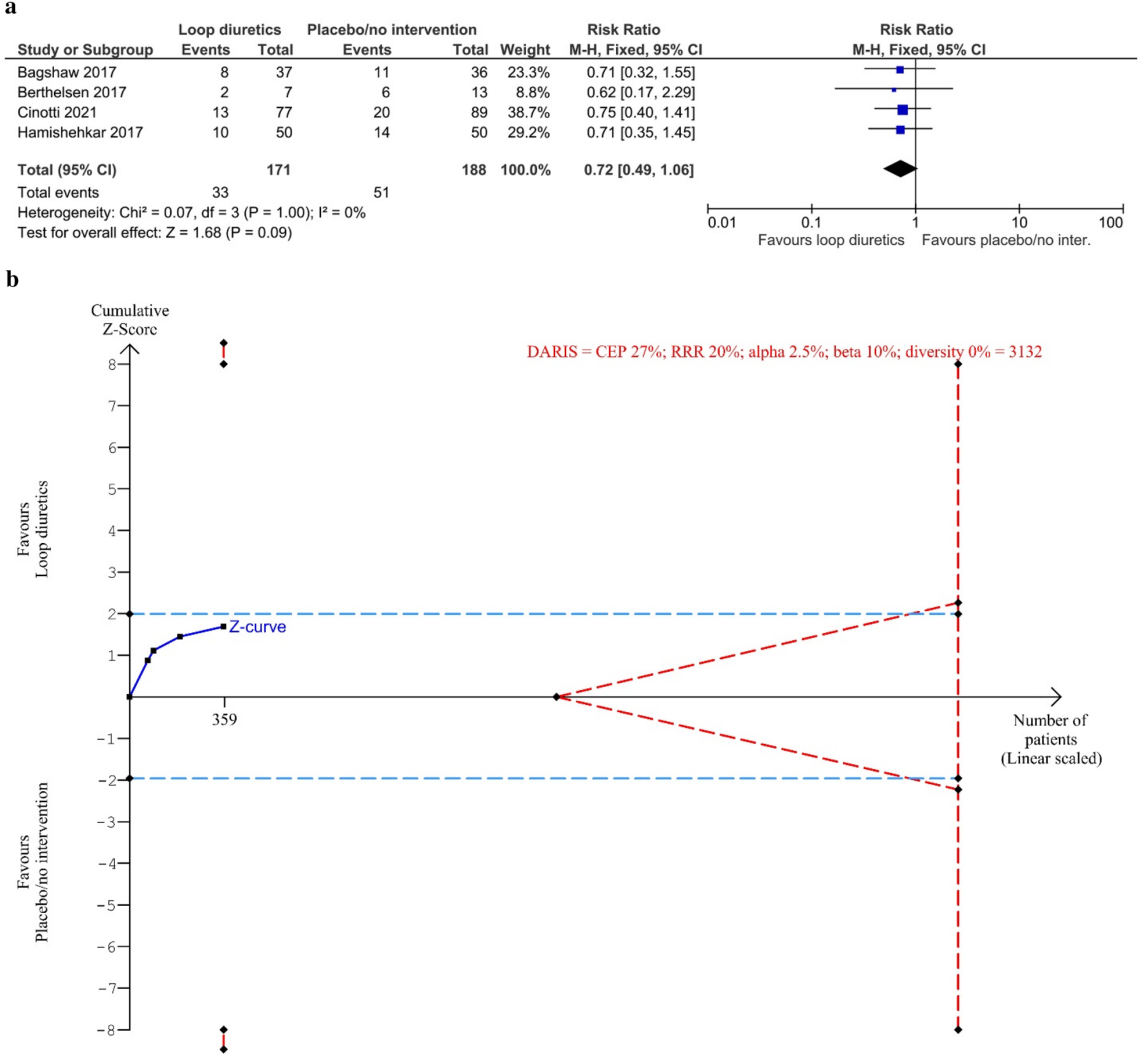
Meta-analysis and TSA for all-cause mortality for loop diuretics vs. placebo/no intervention. **a** Meta-analysis. **b** TSA. The diversity adjusted required information size (DARIS) was calculated according to a mortality proportion in the control group (CEP) of 27%; risk ratio reduction (RRR) of 20% in the experimental intervention group; alpha of 1.7%; a beta of 10% (90% power); and diversity 0%. The DARIS was 3132 participants. The cumulative Z-curve (blue line) did not cross the trial sequential boundaries for benefit or harm or the inner-wedge futility line (red outward sloping red lines) nor the DARIS. The light blue dotted lines show naïve conventional boundaries (alpha 5%)

Four trials reported on all-cause mortality with a follow-up of 28–90 days. The meta-analysis showed no difference between the group treated with loop diuretics vs. placebo/no intervention group (relative risk (RR) 0.72, 95% CI 0.49–1.06; *I*^2^ = 0%; 359 participants, 4 trials; TSA-adjusted CI 0.15–3.48) (Fig. 2). TSA showed that only 11.5% of diversity-adjusted required information size (DARIS) (3132 participants) was accrued and no monitoring boundaries for benefit, harm, or futility were crossed (Fig. 2). Bayes factor for a 20% relative risk reduction was 0.29. Tests for subgroup interaction showed no statistically significant differences (Additional file 1: S6c). The sensitivity analyses assessing incomplete outcome data did not seem to have the potential to influence the result (Additional file 1: S6d). The certainty of evidence was very low (Table 2).

**Table 2:** Summary of Findings with GRADE evaluation for loop diuretics vs. placebo/no intervention

Certainty assessment							No. of patients		Effect		Certainty	Importance
No. of studies	Study design	Risk of Bias	Inconsistency	Indirectness	Imprecission	Other considerations	Loop diuretics	Placebo/no intervention	Relative (95% CI)	Absolute (95% CI)		
All-cause mortality												
4	RCT	Serious^a^	Not serious	Serious^b^	Very serious^c^	None	33/171 (19.3%)	51/188 (27.1%)	RR 0.72 (0.49–1.06)	76 fewer per 1.000 (from 138 fewer to 16 more)	⨁◯◯◯ VERY LOW	CRITICAL
Quality of life—not reported												
–	–	–	–	–	–	–	–	–	–	–	–	CRITICAL
Serious adverse events (SAE)												
6	RCT	Serious^a^	Not serious	Serious^b^	Serious^d^	None	87/230 (37.8%)	116/246 (47.2%)	RR 0.81 (0.66–0.99)	90 fewer per 1.000 (from 160 to 5 fewer)	⨁◯◯◯ VERY LOW	CRITICAL
Plasma concentration of creatinine												
3^e^	RCT	Serious^a^	Not serious	Not serious	Serious^f^	None	0/0	0/0	The trials showed no significant difference in plasma creatinine at longest follow-up		⨁⨁◯◯ LOW	CRITICAL
Proportion of participants without resolution of fluid overload												
2	RCT	Serious^a^	Not serious	Very serious^g^	Very serious^h^	None	4/41 (9.8%)	24/51 (47.1%)	RR 0.22 (0.08–0.58)	367 fewer per 1.000 (from 433 to 198 fewer)	⨁◯◯◯ VERY LOW	CRITICAL
Days on mechanical ventilation												
2^i^	RCT	Serious^a^	Not serious	Serious^b^	Serious^j^	None	0/0	0/0	The trials showed no significant difference in days in mechanical ventilation		⨁◯◯◯ VERY LOW	IMPORTANT
Length of stay in ICU												
2^i^	RCT	Serious^a^	Not serious	Serious^k^	Serious^j^	None	0/0	0/0	The trials showed no significant difference in length of stay in the ICU		⨁◯◯◯ VERY LOW	IMPORTANT
Adverse event not considered serious (AE)												
2	RCT	Serious^a^	Not serious	Serious^l^	Serious^m^	None	71/120 (59.7%)	61/125 (48.8%)	RR 1.23 (0.98–1.55)	112 more per 1.000 (from 10 fewer to 268 more)	⨁◯◯◯ VERY LOW	NOT IMPORTANT

*RCT* randomised clinical trials, *CI* Confidence interval, *RR* Risk ratio

^a^All trials were at overall high risk of bias for this outcome

^b^Variations in experimental intervention and control groups

^c^TSA showed lack of data, because only 11.5% of optimal information size had been reached

^d^TSA showed lack of data, because only 34.7% of optimal information size had been reached

^e^Three trials reported on plasma creatinine. A meta-analysis could not be performed because of unsuitable data (medians and interquartile range or only graphical presentation of data)

^f^The total number of participants were only 193 in the included trials, which is concerning for imprecision

^g^Differences in ICU subpopulation (AKI vs. decompensated heart failure). Resolution of fluid overload is a surrogate outcome

^h^TSA showed lack of data. Only 6.2% of optimal information size had been reached

^i^Data was not suitable for meta-analysis. Both trials found no difference between groups

^j^The total number of participants were only 186 in the included trials, which is concerning for imprecision

^k^The two trials were dissimilar regarding ICU population, control group and length of stay in the ICU

^l^Differences in ICU subpopulation (AKI patients vs. mixed population)

^m^The total number of participants were only 244 in the included trials, which is concerning for imprecision

#### Health-related quality of life

None of the trials reported on health-related quality of life.

#### Serious adverse events

**Fig. 3:**
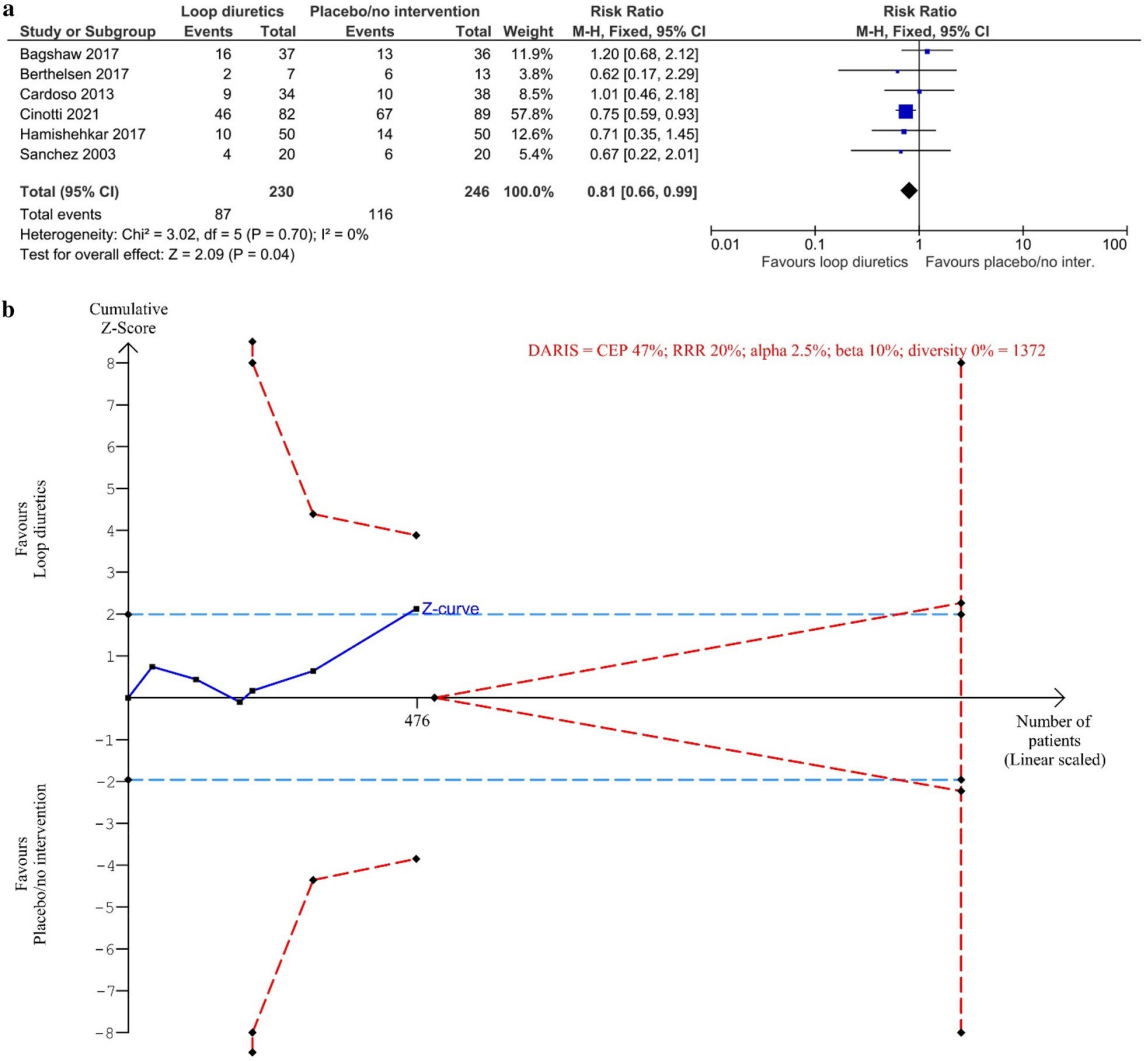
Meta-analysis and TSA on highest event rate of SAEs for loop diuretics vs. placebo/no intervention. **a** Meta-analysis. **b** TSA. The diversity adjusted required information size (DARIS) was calculated according to the proportion of SAEs in the control group (CEP) of 47%; risk ratio reduction (RRR) of 20% in the experimental intervention group; alpha of 1.7%; a beta of 10% (90% power); and diversity 0%. The DARIS was 1372 participants. The cumulative Z-curve (blue line) did not cross the trial sequential boundaries for benefit or harm or the inner-wedge futility line (red outward sloping red lines) nor the DARIS. The light blue dotted lines show naïve conventional boundaries (alpha 5%)

None of the trials reported on the proportion of participants with one or more SAEs. Six trials reported on events we categorised as SAEs [37, 52, 54–57]. We chose to analyse the single SAE with the highest event rate in each trial instead. The meta-analysis showed fewer SAEs in the group treated with loop diuretics vs. placebo/no intervention, but the TSA-adjusted result was not significant (RR 0.81, 95% CI 0.66–0.99; *I*^2^ = 0%; 476 participants; 6 trials; TSA-adjusted CI 0.55–1.20) (Fig. 3). TSA showed that only 34.7% of DARIS (1372 participants) was accrued and no monitoring boundaries for benefit, harm, or futility were crossed (Fig. 3). Bayes factor for a 20% relative risk reduction was = 0.15. Tests for subgroup interaction showed no statistically significant differences (Additional file 1: S6c). The sensitivity analyses assessing incomplete outcome data did not seem to have the potential to influence the result (Additional file 1: S6d). The certainty of evidence was very low (Table 2).

All individual single SAEs and analyses are described in the Supplementary. Meta-analyses were conducted on the following single SAEs: renal replacement therapy (RRT), worsening of AKI, and atrial fibrillation. Meta-analysis showed no difference between the groups treated with loop diuretics vs. placebo/no intervention on RRT (RR 1.12, 95% CI 0.67–1.88; *I*^2^ = 0%; 299 participants, 4 trials); worsening of AKI (RR 0.86, 95% CI 0.63–1.18; *I*^2^ = 29%; 316 participants, 3 trials); and atrial fibrillation (RR 0.71, 95% CI 0.39–1.31; *I*^2^ = 0%; 264 participants, 3 trials).

#### Adverse events not considered serious

None of the trials reported on the proportion of participants with one or more adverse events not considered serious. Two trials reported on individual AEs [52, 54]. The single AE with the highest event proportion in each trial was analysed instead. Meta-analysis showed no difference in occurrence of AEs in the group treated with loop diuretics vs. placebo/no intervention (RR 1.23, 95% CI 0.98–1.55; I^2^ = 43%; 245 participants; 2 trials; TSA-adjusted CI 0.28–5.56). TSA showed that only 6.7% of DARIS (3645 participants) was accrued and no monitoring boundaries for benefit, harm or futility were crossed (Additional file 1: S6b). Sensitivity analyses assessing incomplete outcome data did not seem to have the potential to influence the result (Additional file 1: S6d). Certainty of evidence was very low (Table 2).

All single AEs were only reported once, thus meta-analyses could not be conducted (Additional file 1: S6e).

#### Plasma concentration of creatinine

Three trials reported on creatinine using medians and IQR [37, 52, 56]. The individual trials showed no difference between the group treated with loop diuretics vs. placebo/no intervention. The data were not in a format suitable for meta-analysis. Certainty of evidence was low (Table 2).

#### Participants without resolution of fluid overload

Two trials [37, 55] reported on resolution of fluid overload. The meta-analysis showed that the proportion of participants without resolution of fluid overload was smaller in the group treated with loop diuretic vs. placebo/no intervention, but this was not confirmed with TSA (RR 0.22, 95% CI 0.08–0.58; I^2^ = 0%; 92 participants; 2 trials; TSA-adjusted CI 0.00–11.80). TSA showed that only 6.2% of DARIS (1487 participants) was accrued and no monitoring boundaries for benefit, harm, or futility were crossed (Additional file 1: S6b). Certainty of evidence was very low (Table 2).

#### Number of days on mechanical ventilation and length of stay in the ICU

Two trials [37, 54] reported on these two outcomes using medians and IQR and were not suitable for meta-analysis. Both trials found no difference between groups. Certainty of evidence was very low (Table 2).

#### Plasma concentration of serum sodium, potassium, and chloride concentrations

Two trials [37, 52] reported on sodium and potassium concentrations. The data was not suitable for meta-analysis. One trial [52] found no difference on potassium between the group treated with loop diuretics vs. placebo/no intervention but found that sodium was higher in the group treated with loop diuretics. No data on chloride was available. The other trial [37] found no difference in potassium, sodium, and chloride concentrations between the group treated with loop diuretics vs. placebo/no intervention.

### Results for loop diuretics (furosemide) vs. another loop diuretic (piretanide or ethacrynic acid)

Two trials compared loop diuretic vs. another loop diuretic (260 participants) [58, 59]. Both trials included patients from cardiac ICUs. One trial with 12 participants tested furosemide vs. piretanide [58]. The other trial investigated furosemide vs. ethacrynic acid in 248 participants [59]. Two meta-analyses were possible for this comparison: plasma concentration of sodium (MD − 1.86 mmol/L; 95% CI − 6.27–2.54; *I*^2^ = 71%; 260 participants; 2 trials) and potassium (MD − 0.04 mmol/L; 95% CI − 0.16–0.08; *I*^2^ = 0%; 260 participants; 2 trials), showing no differences. The analyses and a detailed narrative description of the outcomes in the two trials is presented in the Additional file 1: S7b, S7c. S7d and S7e.

### Results for loop diuretic (furosemide) vs. another type of diuretic (acetazolamide or tolvaptan)

Two trials compared loop diuretics vs. another type of diuretic (58 participants) [53, 60]. One trial included mixed ICU patients and investigated the effects of furosemide vs. acetazolamide over a study time of just 6 h [53]. The other trial included patients with decompensated hearth failure in a medical ICU investigating furosemide vs. tolvaptan for up to 96 h [60]. No meta-analyses could be performed on any outcomes. Detailed narrative description of the outcomes in the two trials is in the Additional file 1: S8b, S8c, and S8d.

## Discussion

In this systematic review ten trials were included involving six types of diuretics. Six trials compared a loop diuretic (furosemide or torsemide) with placebo/no intervention. Our main results are based on this comparison in adult ICU patients with fluid overload.

Furosemide was tested against another loop diuretic (piretanide or ethacrynic acid) in two trials and against two different types of diuretics (acetazolamide or tolvaptan) in two other trials. Primary and secondary outcomes of these trials could not be meta-analysed.

We found no difference in mortality when comparing loop diuretics vs. placebo/no intervention in ICU patients with fluid overload, but there seemed to be fewer SAEs in those treated with loop diuretics in the meta-analysis; however, the TSA-adjusted-CI crossed 1.0 (no effect) and the DARIS was far from reached. The proportion of participants without resolution of fluid overload was lower in the group treated with loop diuretic; again, the TSA did not confirm this. Effects on plasma concentrations of electrolytes and AEs were inconclusive. Health-related quality of life, length of stay in ICU, time on mechanical ventilation, and plasma concentrations of creatinine could not be analysed due to lack of data. All outcomes were adjudicated to be at low or very low certainty of evidence or no evidence at all.

## Strengths and limitations

The strength of this systematic review of RCTs is the methodological quality, which included adhering to our pre-published protocol [26] and using the recommendations of the Cochrane Handbook on interventions [27]. We assessed risk of bias using the ROB2-tool [33] and followed the eight steps procedure by Jakobsen and co-workers [28]. We assessed the certainty of evidence with GRADE [29, 50] and reported the review as recommended by PRISMA [30].

### Limitations

We only identified few and small trials, and all outcomes were at high risk of bias. Clinical heterogeneity between the trials was high; fluid overload was not defined in all trials and resolution of fluid overload was sparsely reported.

Fluid overload was defined as a percentage calculated from fluid balance and body weight on admission to the ICU or according to ideal body weight or by clinical signs of water retention (oedema, pulmonary crepitations, elevated jugular venous pressure, hepatomegaly). We also included RCTs with loop diuretics in ICU patients with AKI and acute heart failure even if fluid overload was not defined. These conditions are associated with fluid overload and we considered these groups of patients to have fluid overload when entering a trial of protocolised diuretic therapy [26]. We did that to assess all relevant RCTs in the field, but it is also a limitation due to an uncertainty of the degree of fluid overload.

Furthermore, the outcomes in the included trials were heterogenic making comparisons difficult. The experimental and the control regiments were insufficiently reported in several trials. The use of diuretics as escape or protocol violations in trials with placebo or no diuretics as control group hampers the interpretation further. Moreover, we only looked at a single loop diuretic as experimental intervention. Combinations of different loop diuretics need to be assessed in other systematic reviews.

### Current results in relation to previous reviews

Fluid overload in ICU patients is common and a risk factor for death [66]. This review assessed the existing evidence of treating fluid overload with loop diuretics in ICU patients. No systematic reviews on treatment of fluid overload with loop diuretics vs. a control group in the ICU setting has been performed before. Two former systematic reviews focusing on liberal fluid therapy vs. conservative fluid therapy/de-resuscitation in ICU found diverging results. A review from 2014 [67], which pooled observational data together with data from RCTs, found that non survivors had a more positive fluid balance compared to survivors. Restrictive fluid management was associated with a lower mortality compared to liberal fluid management. Only some of the included trials involved diuretics. Another review from 2017 [68] focussed on conservative or de-resuscitative fluid strategies in adults and children with acute respiratory distress syndrome or sepsis in the post-resuscitation phase of critical illness. This meta-analysis of RCTs found no difference in mortality but a conservative or de-resuscitative strategy resulted in more ventilator-free days and shorter length of ICU stay compared with liberal fluid strategy or standard of care. Only few of the included trials involved diuretics. A systematic review from 2018 with pooled data from both observational studies and RCTs, assessed continuous infusion vs. intermittent bolus injection of furosemide in ICU patients [69]. This review found a larger diuretic effect for patients treated with continuous infusion compared to bolus injection. No differences in mortality or renal function were found.

### Clinical implications and perspectives

Besides the fundamental lack of data, we identified numerous factors in the existing literature that hampers the interpretation of our results, for example the lack of a standardised definition of fluid overload and how to assess it. The trials investigating the effect of diuretics seldomly described or defined fluid overload and quantified it. The effect of diuretic therapy is likely influenced by the severity of fluid overload and the differing description makes it difficult to generalise and compare results. The trials often report urine output, fluid balance, or weight changes in a predefined timeframe but information about resolution of fluid overload was rarely reported. When assessing data on mortality it is important to know if fluid overload is removed or mitigated by the intervention/treatment. This would make the assessment of mortality and other patient important outcomes more reliable.

The use of diuretics in the ICU patients appears safe due to fewer SAEs in the group treated with loop diuretics and no difference in single SAEs between groups. Timing of prescribing diuretics might have an impact on development of SAEs, which is not covered in this review.

Early prescription of diuretics, while the patient receives vasoactive therapy may reduce sodium chloride (NaCl) and water accumulation or minimise further accumulation which might reduce the adverse effects of fluid overload. It can be argued that later prescription of diuretics in the recovery phase is safer. The patient will be without vasoactive drugs and the risk of hypoperfusion is less. The evidence on this subject is sparce and conflicting [70–72]. The timing of prescribing diuretics in the ICU population with fluid overload would be relevant to investigate in a future RCT.

Patients with sepsis and septic shock have an increased risk of developing fluid overload following fluid resuscitation and about 40% receive diuretics during their ICU stay [73, 74]. This makes the debate of restrictive vs. liberal fluid therapy important. Focus on avoiding fluid administration when the perfusion is adequate, even if vasopressors are needed, and if the perfusion is inadequate, it is important to assess if fluid responsiveness is likely before fluid administration [75]. This could be an approach to minimise the risk of severe fluid overload.

It is important to keep in mind that the sodium administration to ICU patients often are much higher than normal dietary intake due to fluid therapy, nutrition, and isotonic sodium containing fluids used as drug dissolvents [1]. This is an important cofactor in development of fluid overload. Reducing sodium intake using hypotonic or low sodium solutions as maintenance fluid, dissolve medicine in dextrose 5% or glucose 5%, and convert to oral medication when possible, the sodium load can be minimised and the associated water retention [1]. Moreover, reduced sodium intake might reduce the risk of hypernatremia. Loop diuretics induces lager free water excretion compared to sodium excretion and can contribute to development of hypernatremia which is associated with increased mortality [76, 77].

Diuretic resistance can be a challenge in the ICU. Infusion of loop diuretic instead of bolus injections and combination therapy with loop diuretic and thiazides or carbon anhydrase inhibitors might increase the diuretic output but there is a risk of increased adverse effects [78].

It is still unclear if active de-resuscitation with loop diuretics in adult ICU patients with fluid overload will improve patient-important outcomes. A general accepted definition of fluid overload and resolution of fluid overload is missing. No gold standard method of measuring fluid status and no general accepted definition of fluid overload exist. We suggest defining fluid overload as > 5% increase in body water assessed according to fluid balances, changes in body weight, and clinical examination. Resolution of fluid overload should be assessed the same way. The surrogate outcomes are too imprecise when used alone. The weight on admission to the ICU might not represent the patient’s habitual weight and during critical illness muscle mass is lost which makes body weight an imprecise measure. Fluid balances from the ICU will be imprecise, because the time in the hospital before referral to the ICU is not accounted for. Severely ill patients might have an affected fluid balance already on admission to the hospital, which are not reflected in the fluid charts. Clinical examination (oedema, lung ultrasound, radiologic findings, and other measures) is imprecise to assess the degree of fluid but it is needed to support, correct or to confirm the findings from development in body weight and fluid balance. A discussion of all the surrogate measurements for assessing fluid status is important but outside the scope of this review.

In the presence of insufficient evidence for the use of diuretics, it should be restricted to patients who may benefit the most based on physiological and observational data. Patients with sodium and water accumulation with associated respiratory insufficiency without other clear causes might benefit the most from diuretics. Retrospective data suggest that loop diuretics in patients with acute respiratory distress syndrome reduce mortality [79].

Large RCTs at low risk of bias are needed before definitive conclusions can be made on treatment of fluid overload with diuretics in adult ICU patients.

## Conclusions

The evidence is very uncertain about the effect of loop diuretics on mortality and serious adverse events in adult ICU patients with fluid overload. Loop diuretics may reduce the occurrence of these outcomes, but large randomised placebo-controlled trials at low risk of bias are needed.

## Supplementary Information

**Additional file 1.** **S1.** PRISMA checklist. **S2.** Search strategy. **S3.** Post hoc subgroup analysis for the comparison of loop diuretics vs. placebo/no intervention. **S4.** Detailed characteristics of included trials. **S5.** Overall risk of bias for all included trials. **S6.** Comparison: loop diuretics vs. placebo/no intervention. **S6a.** Risk of bias of all outcomes. **S6b.** Meta-analyses and TSA. **S6c.** Subgroup analyses. **S6d.** Sensitivity analyses. **S6e.** Reported SAEs and AEs. **S7.** Comparison: loop diuretics vs. another loop diuretic. **S7a.** Risk of bias. **S7b.** Meta-analyses. **S7c.** Narrative description of the results. **S7d.** Reported SAEs and AEs. **S7e.** Summary of findings. **S8.** Comparison: loop diuretics vs. another type of diuretic. **S8a.** Risk of bias. **S8b.** Narrative description of the results. **S8c.** Reported SAEs and AEs. **S8d.** Summary of findings.

## Data Availability

All data analysed in this study are included in the published article and in the Additional file material.

## Abbreviations

ICU: Intensive care unit
TSA: Trial Sequential Analysis
PRISMA: Preferred reporting items for systematic reviews and meta-analysis
GRADE: Grading of recommendations, assessments, developments and evaluations
AKI: Acute kidney injury
RCT: Randomised clinical trials
SAE: Serious adverse event
ICH-GCP: Good Clinical Practice Guideline of the International Conference on Harmonization
AE: Adverse event
CNKI: China National Knowledge Infrastructure
FDA: Food and drug administration
EMA: European medicines agency
RR: Risk ratios
CI: Confidence interval
MD: Mean difference
ALI: Acute lung injury
ARDS: Acute respiratory distress syndrome
IQR: Interquartile range
DARIS: Diversity-adjusted required information size
RRT: Renal replacement therapy
