# Supplementary material for: Loop diuretics in adult intensive care patients with fluid overload: a systematic review of randomised clinical trials with meta-analysis and trial sequential analysis
Source: Ann Intensive Care. 2022 Jun 13;12:52. doi: 10.1186/s13613-022-01024-6 (PMC9192894; doi:10.1186/s13613-022-01024-6)
Supplement: Supplementary file 1 — Additional file 1. S1. PRISMA checklist. S2. Search strategy. S3. Post hoc subgroup analysis for the comparison of loop diuretics vs. placebo/no intervention. S4. Detailed characteristics of included trials. S5. Overall risk of bias for all included trials. S6. Comparison: loop diuretics vs. placebo/no intervention. S6a. Risk of bias of all outcomes. S6b. Meta-analyses and TSA. S6c. Subgroup analyses. S6d. Sensitivity analyses. S6e. Reported SAEs and AEs. S7. Comparison: loop diuretics vs. another loop diuretic. S7a. Risk of bias. S7b. Meta-analyses. S7c. Narrative description of the results. S7d. Reported SAEs and AEs. S7e. Summary of findings. S8. Comparison: loop diuretics vs. another type of diuretic. S8a. Risk of bias. S8b. Narrative description of the results. S8c. Reported SAEs and AEs. S8d. Summary of findings. [file 13613_2022_1024_MOESM1_ESM.docx]

**SUPPLEMENTARY MATERIAL**

This supplementary material has been provided by the authors to give readers additional information about their work.

Supplement to:

**Wichmann S, Barbateskovic M, Liang N, Itenov TS, Berthelsen RE, Lindschou J, Perner A, Gluud C, Bestle MH. Loop diuretics in adult intensive care patients with fluid overload: a systematic review of randomised clinical trials with meta-analysis and Trial Sequential Analysis.**

Correspondence: Wichmann S, Department of Anaesthesiology, Copenhagen University Hospital – North Zealand, Denmark, e-mail: [sine.wichmann@regionh.dk](mailto:sine.wichmann@regionh.dk)

**Electronic Supplement Material for**

**Loop diuretics in adult intensive care patients with fluid overload: a systematic review of randomised clinical trials with meta-analysis and Trial Sequential Analysis**

**Content**

[**S1. PRISMA checklist** 3](#_Toc103946990)

[**S2. Search strategy** 6](#_Toc103946991)

[**S3. Post hoc subgroup analysis for the comparison of loop diuretics vs placebo/no intervention** 13](#_Toc103946992)

[**S4. Detailed characteristics of included trials** 14](#_Toc103946993)

[**S5. Overall risk of bias for all included trials** 22](#_Toc103946994)

[**S6. Comparison: loop diuretics vs placebo/no intervention** 25](#_Toc103946995)

[**S6a. Risk of bias of all outcomes** 25](#_Toc103946996)

[**S6b. Meta-analyses and TSA** 32](#_Toc103946997)

[**S6c. Subgroup analyses** 35](#_Toc103946998)

[**S6d. Sensitivity analyses** 42](#_Toc103946999)

[**S6e. Reported SAEs and AEs** 45](#_Toc103947000)

[**S7. Comparison: loop diuretics vs another loop diuretic** 47](#_Toc103947001)

[**S7a. Risk of bias** 47](#_Toc103947002)

[**S7b. Meta-analyses** 49](#_Toc103947003)

[**S7c. Narrative description of the results** 50](#_Toc103947004)

[**S7d. Reported SAEs and AEs** 51](#_Toc103947005)

[**S7e. Summary of findings** 52](#_Toc103947006)

[**S8. Comparison: loop diuretics vs another type of diuretic** 53](#_Toc103947007)

[**S8a. Risk of bias** 53](#_Toc103947008)

[**S8b. Narrative description of the results** 56](#_Toc103947009)

[**S8c. Reported SAEs and AEs** 57](#_Toc103947010)

[**S8d. Summary of findings** 58](#_Toc103947011)

# **S1. PRISMA checklist**

**Table S1.** Prisma checklist

| **Section and Topic** | **Item #** | **Checklist item** | **Location where item is reported** |
| --- | --- | --- | --- |
| **TITLE** | | |  |
| Title | 1 | Identify the report as a systematic review. | 1 |
| **ABSTRACT** | | |  |
| Abstract | 2 | See the PRISMA 2020 for Abstracts checklist. | 3 |
| **INTRODUCTION** | | |  |
| Rationale | 3 | Describe the rationale for the review in the context of existing knowledge. | 4 |
| Objectives | 4 | Provide an explicit statement of the objective(s) or question(s) the review addresses. | 5 |
| **METHODS** | | |  |
| Eligibility criteria | 5 | Specify the inclusion and exclusion criteria for the review and how studies were grouped for the syntheses. | 6 |
| Information sources | 6 | Specify all databases, registers, websites, organisations, reference lists and other sources searched or consulted to identify studies. Specify the date when each source was last searched or consulted. | 6-7 + supplementary |
| Search strategy | 7 | Present the full search strategies for all databases, registers and websites, including any filters and limits used. | 6-7 + supplementary |
| Selection process | 8 | Specify the methods used to decide whether a study met the inclusion criteria of the review, including how many reviewers screened each record and each report retrieved, whether they worked independently, and if applicable, details of automation tools used in the process. | 7 |
| Data collection process | 9 | Specify the methods used to collect data from reports, including how many reviewers collected data from each report, whether they worked independently, any processes for obtaining or confirming data from study investigators, and if applicable, details of automation tools used in the process. | 7 |
| Data items | 10a | List and define all outcomes for which data were sought. Specify whether all results that were compatible with each outcome domain in each study were sought (e.g. for all measures, time points, analyses), and if not, the methods used to decide which results to collect. | 6-7 |
|  | 10b | List and define all other variables for which data were sought (e.g. participant and intervention characteristics, funding sources). Describe any assumptions made about any missing or unclear information. | 7 |
| Study risk of bias assessment | 11 | Specify the methods used to assess risk of bias in the included studies, including details of the tool(s) used, how many reviewers assessed each study and whether they worked independently, and if applicable, details of automation tools used in the process. | 8 |
| Effect measures | 12 | Specify for each outcome the effect measure(s) (e.g. risk ratio, mean difference) used in the synthesis or presentation of results. | 8 |
| Synthesis methods | 13a | Describe the processes used to decide which studies were eligible for each synthesis (e.g. tabulating the study intervention characteristics and comparing against the planned groups for each synthesis (item #5)). | 6- 7 |
|  | 13b | Describe any methods required to prepare the data for presentation or synthesis, such as handling of missing summary statistics, or data conversions. | 8-10 |
|  | 13c | Describe any methods used to tabulate or visually display results of individual studies and syntheses. | 8-10 |
|  | 13d | Describe any methods used to synthesize results and provide a rationale for the choice(s). If meta-analysis was performed, describe the model(s), method(s) to identify the presence and extent of statistical heterogeneity, and software package(s) used. | 8-10 |
|  | 13e | Describe any methods used to explore possible causes of heterogeneity among study results (e.g. subgroup analysis, meta-regression). | 9 |
|  | 13f | Describe any sensitivity analyses conducted to assess robustness of the synthesized results. | 9 |
| Reporting bias assessment | 14 | Describe any methods used to assess risk of bias due to missing results in a synthesis (arising from reporting biases). | 8 |
| Certainty assessment | 15 | Describe any methods used to assess certainty (or confidence) in the body of evidence for an outcome. | 11 |
| **RESULTS** | | |  |
| Study selection | 16a | Describe the results of the search and selection process, from the number of records identified in the search to the number of studies included in the review, ideally using a flow diagram. | 11 + Fig 1 + supplementary |
|  | 16b | Cite studies that might appear to meet the inclusion criteria, but which were excluded, and explain why they were excluded. | Fig. 1 |
| Study characteristics | 17 | Cite each included study and present its characteristics. | Table 1 + supplementary |
| Risk of bias in studies | 18 | Present assessments of risk of bias for each included study. | 12 + supplementary |
| Results of individual studies | 19 | For all outcomes, present, for each study: (a) summary statistics for each group (where appropriate) and (b) an effect estimate and its precision (e.g. confidence/credible interval), ideally using structured tables or plots. | 12-16 + supplementary |
| Results of syntheses | 20a | For each synthesis, briefly summarise the characteristics and risk of bias among contributing studies. | Table 2 + supplementary |
|  | 20b | Present results of all statistical syntheses conducted. If meta-analysis was done, present for each the summary estimate and its precision (e.g. confidence/credible interval) and measures of statistical heterogeneity. If comparing groups, describe the direction of the effect. | 12-16 + Fig 2 + Fig 3 + supplementary |
|  | 20c | Present results of all investigations of possible causes of heterogeneity among study results. | 12-13 + supplementary |
|  | 20d | Present results of all sensitivity analyses conducted to assess the robustness of the synthesized results. | 12-14 + supplementary |
| Reporting biases | 21 | Present assessments of risk of bias due to missing results (arising from reporting biases) for each synthesis assessed. | 12 + supplementary |
| Certainty of evidence | 22 | Present assessments of certainty (or confidence) in the body of evidence for each outcome assessed. | 12-15 + Table 2 + supplementary |
| **DISCUSSION** | | |  |
| Discussion | 23a | Provide a general interpretation of the results in the context of other evidence. | 18-22 |
|  | 23b | Discuss any limitations of the evidence included in the review. | 17-18 |
|  | 23c | Discuss any limitations of the review processes used. | 17-18 |
|  | 23d | Discuss implications of the results for practice, policy, and future research. | 18-22 |
| **OTHER INFORMATION** | | |  |
| Registration and protocol | 24a | Provide registration information for the review, including register name and registration number, or state that the review was not registered. | 3 + 5 |
|  | 24b | Indicate where the review protocol can be accessed, or state that a protocol was not prepared. | 5 |
|  | 24c | Describe and explain any amendments to information provided at registration or in the protocol. | 10 + supplementary |
| Support | 25 | Describe sources of financial or non-financial support for the review, and the role of the funders or sponsors in the review. | 23-24 |
| Competing interests | 26 | Declare any competing interests of review authors. | 24 |
| Availability of data, code and other materials | 27 | Report which of the following are publicly available and where they can be found: template data collection forms; data extracted from included studies; data used for all analyses; analytic code; any other materials used in the review. | supplementary* |

* All analyses are presented in supplementary. A data collection form is available upon request from corresponding author.

*From:*  Page MJ, McKenzie JE, Bossuyt PM, Boutron I, Hoffmann TC, Mulrow CD, et al. The PRISMA 2020 statement: an updated guideline for reporting systematic reviews. BMJ 2021;372:n71. doi: 10.1136/bmj.n71

For more information, visit: <http://www.prisma-statement.org/>

# **S2. Search strategy**

**Cochrane Central Register of Controlled Trials (CENTRAL)**

Searched May 25^th^, 2020 Records identified: 2215

Updated search April 13^th^, 2021 Records identified: 2372

#1 MeSH descriptor: [Furosemide] explode all trees

#2 MeSH descriptor: [Torsemide] explode all trees

#3 MeSH descriptor: [Bumetanide] explode all trees

#4 MeSH descriptor: [Ethacrynic Acid] explode all trees

#5 MeSH descriptor: [Diuretics] explode all trees

#6 MeSH descriptor: [Fluid Therapy] explode all trees and with qualifier(s): [adverse effects - AE]

#7 MeSH descriptor: [Water-Electrolyte Balance] explode all trees

#8 MeSH descriptor: [Edema] explode all trees

#9 ((furosemide or torsemide or bumetanide or ethacrynic acid or azosemide or diuretic*)):ti,ab,kw

#10 (((fluid overload or hyperhydration or overhydration or positive fluid balance or hydration* or edema or water electrolyte imbalance) and (furosemide or torsemide or bumetanide or ethacrynic acid or azosemide or diuretic*))):ti,ab,kw

#11 #1 or #2 or #3 or #4 or #5 or #6 or #7 or #8 or #9 or #10

#12 MeSH descriptor: [Critical Illness] explode all trees

#13 MeSH descriptor: [Critical Care] explode all trees

#14 MeSH descriptor: [Intensive Care Units] explode all trees

#15 ((critically ill or acutely ill or intensive care or critical care or ICU*)):ti,ab,kw

#16 MeSH descriptor: [Shock] explode all trees

#17 (shock):ti,ab,kw

#18 MeSH descriptor: [Acute Lung Injury] explode all trees

#19 ((acute lung injury or respiratory failure)):ti,ab,kw

#20 MeSH descriptor: [Respiratory Distress Syndrome, Adult] explode all trees

#21 MeSH descriptor: [Respiratory Insufficiency] explode all trees

#22 ((respiratory distress syndrome or ARDS or respiratory failure)):ti,ab,kw

#23 MeSH descriptor: [Multiple Trauma] explode all trees

#24 ((severe trauma or multiple trauma)):ti,ab,kw

#25 ((trauma and (ICU* or intensive care))):ti,ab,kw

#26 MeSH descriptor: [Sepsis] explode all trees

#27 MeSH descriptor: [Shock, Septic] explode all trees

#28 ((sepsis or septic shock)):ti,ab,kw

#29 MeSH descriptor: [Liver Failure, Acute] explode all trees

#30 ((acute hepatic failure or fulminating hepatic failure or renal failure or acute tubular necrosis)):ti,ab,kw

#31 MeSH descriptor: [Acute Kidney Injury] explode all trees

#32 ((acute kidney failure or acute renal injuries)):ti,ab,kw

#33 MeSH descriptor: [Pulmonary Edema] explode all trees

#34 (pulmonary edema):ti,ab,kw

#35 #12 or #13 or #14 or #15 or #16 or #17 or #18 or #19 or #20 or #21 or #22 or #23 or #24 or #25 or #26 or #27 or #28 or #29 or #30 or #31 or #32 or #33 or #34

#36 #11 and #35

**MEDLINE (OvidSP)**

Searched May 25^th^, 2020 Records identified: 1076

Updated search April 13^th^, 2021 Records identified: 1126

1. exp Furosemide/

2. exp Torsemide/

3. exp Bumetanide/

4. exp Ethacrynic Acid/

5. exp Diuretics/

6. exp Fluid Therapy/ae [Adverse Effects]

7. exp Water-Electrolyte Balance/

8. exp Edema/

9. (furosemide or torsemide or bumetanide or ethacrynic acid or azosemide or diuretic*).tw.

10. ((fluid overload or hyperhydration or overhydration or positive fluid balance or hydration* or edema or water electrolyte imbalance) and (furosemide or torsemide or bumetanide or ethacrynic acid or azosemide or diuretic*)).tw.

11. 1 or 2 or 3 or 4 or 5 or 6 or 7 or 8 or 9 or 10

12. exp Critical Illness/

13. exp Critical Care/

14. exp Intensive Care Units/

15. (critically ill or acutely ill or intensive care or critical care or ICU*).tw.

16. exp Shock/

17. shock.tw.

18. exp Acute Lung Injury/

19. (acute lung injury or respiratory failure).tw.

20. exp Respiratory Distress Syndrome, Adult/

21. exp Respiratory Insufficiency/

22. (respiratory distress syndrome or ARDS or respiratory failure).tw.

23. exp Multiple Trauma/

24. (severe trauma or multiple trauma).tw.

25. (trauma and (ICU* or intensive care)).tw.

26. exp Sepsis/

27. exp Shock, Septic/

28. (sepsis or septic shock).tw.

29. exp Liver Failure, Acute/

30. (acute hepatic failure or fulminating hepatic failure or renal failure or acute tubular necrosis).tw.

31. exp Acute Kidney Injury/

32. (acute kidney failure or acute renal injuries).tw.

33. exp Pulmonary Edema/

34. pulmonary edema.tw.

35. 12 or 13 or 14 or 15 or 16 or 17 or 18 or 19 or 20 or 21 or 22 or 23 or 24 or 25 or 26 or 27 or 28 or 29 or 30 or 31 or 32 or 33 or 34

36. 11 and 35

37. randomized controlled trial.pt.

38. controlled clinical trial.pt.

39. randomized.ab.

40. placebo.ab.

41. clinical trial.sh.

42. randomly.ab.

43. trial.ti.

44. 37 or 38 or 39 or 40 or 41 or 42 or 43

45. exp animals/ not humans.sh.

46. 44 not 45

47. 36 and 46

**PubMed**

Searched May 25^th^, 2020 Records identified: 426

Updated search April 13^th^, 2021 Records identified: 452

(((((((((((Furosemide[MeSH Terms]) OR (Torsemide[MeSH Terms])) OR (Bumetanide[MeSH Terms])) OR (Ethacrynic Acid[MeSH Terms])) OR (Diuretics[MeSH Terms])) OR (Water-Electrolyte Balance[MeSH Terms])) OR (Edema[MeSH Terms])) OR ((furosemide[Text Word] OR torsemide[Text Word] OR bumetanide[Text Word] OR ethacrynic acid[Text Word] OR azosemide[Text Word] OR diuretic*)[Text Word])) OR (((fluid overload[Text Word] OR hyperhydration[Text Word] OR overhydration[Text Word] OR positive fluid balance[Text Word] OR hydration*[Text Word] OR edema[Text Word] OR water electrolyte imbalance)[Text Word] AND (furosemide[Text Word] OR torsemide[Text Word] OR bumetanide[Text Word] OR ethacrynic acid[Text Word] OR azosemide[Text Word] OR diuretic*))[Text Word])) AND ((((((((((((((((((((((Critical Illness[MeSH Terms]) OR (Critical Care[MeSH Terms])) OR (Intensive Care Units[MeSH Terms])) OR (Shock[MeSH Terms])) OR (Acute Lung Injury[MeSH Terms])) OR (Respiratory Distress Syndrome, Adult[MeSH Terms])) OR (Respiratory Insufficiency[MeSH Terms])) OR (Multiple Trauma[MeSH Terms])) OR (Sepsis[MeSH Terms])) OR (Shock, Septic[MeSH Terms])) OR (Liver Failure, Acute[MeSH Terms])) OR (Acute Kidney Injury[MeSH Terms])) OR (Pulmonary Edema[MeSH Terms])) OR ((critically ill[Text Word] OR acutely ill[Text Word] OR intensive care[Text Word] OR critical care[Text Word] OR ICU*)[Text Word])) OR (shock[Text Word])) OR ((acute lung injury[Text Word] OR respiratory failure)[Text Word])) OR ((severe trauma[Text Word] OR multiple trauma)[Text Word])) OR ((trauma[Text Word] AND (ICU*[Text Word] OR intensive care))[Text Word])) OR ((sepsis[Text Word] OR septic shock)[Text Word])) OR ((acute hepatic failure[Text Word] OR fulminating hepatic failure[Text Word] OR renal failure[Text Word] OR acute tubular necrosis)[Text Word])) OR ((acute kidney failure[Text Word] OR acute renal injuries)[Text Word])) OR (pulmonary edema[Text Word]))) AND (((((((((((randomized controlled trial[Publication Type]) OR controlled clinical trial[Publication Type]) OR randomized[Title/Abstract]) OR placebo[Title/Abstract]) OR clinical trials[MeSH Terms]) OR randomly[Title/Abstract]) OR trial[Title])) NOT ((animals[MeSH Terms]) NOT humans[MeSH Terms])))))

**EMBASE (OvidSP)**

Searched May 25^th^, 2020 Records identified: 1434

Updated search April 13^th^, 2021 Records identified: 1522

1. *furosemide/

2. *torasemide/

3. *bumetanide/

4. *etacrynic acid/

5. *diuretic agent/

6. *fluid therapy/ae [Adverse Drug Reaction]

7. *electrolyte balance/

8. *edema/

9. (furosemide or torsemide or bumetanide or ethacrynic acid or azosemide or diuretic*).tw.

10. ((fluid overload or hyperhydration or overhydration or positive fluid balance or hydration* or edema or water electrolyte imbalance) and (furosemide or torsemide or bumetanide or ethacrynic acid or azosemide or diuretic*)).tw.

11. 1 or 2 or 3 or 4 or 5 or 6 or 7 or 8 or 9 or 10

12. exp critical illness/

13. exp intensive care/

14. exp intensive care unit/

15. (critically ill or acutely ill or intensive care or critical care or ICU*).tw.

16. *shock/

17. shock.tw.

18. *acute lung injury/

19. (acute lung injury or respiratory failure).tw.

20. *adult respiratory distress syndrome/

21. *respiratory failure/

22. (respiratory distress syndrome or ARDS or respiratory failure).tw.

23. *multiple trauma/

24. (severe trauma or multiple trauma).tw.

25. (trauma and (ICU* or intensive care)).tw.

26. *sepsis/

27. *septic shock/

28. (sepsis or septic shock).tw.

29. *acute liver failure/

30. (acute hepatic failure or fulminating hepatic failure or renal failure or acute tubular necrosis).tw.

31. *acute kidney failure/

32. (acute kidney failure or acute renal injuries).tw.

33. *lung edema/

34. pulmonary edema.tw.

35. 12 or 13 or 14 or 15 or 16 or 17 or 18 or 19 or 20 or 21 or 22 or 23 or 24 or 25 or 26 or 27 or 28 or 29 or 30 or 31 or 32 or 33 or 34

36. 11 and 35

37. CROSSOVER PROCEDURE.sh.

38. DOUBLE-BLIND PROCEDURE.sh.

39. SINGLE-BLIND PROCEDURE.sh.

40. (crossover* or cross over*).ti,ab.

41. placebo*.ti,ab.

42. (doubl* adj blind*).ti,ab.

43. allocat*.ti,ab.

44. trial.ti.

45. RANDOMIZED CONTROLLED TRIAL.sh.

46. random*.ti,ab.

47. 37 or 38 or 39 or 40 or 41 or 42 or 43 or 44 or 45 or 46

48. (exp animal/ or exp invertebrate/ or animal.hw. or nonhuman/) not (exp human/ or human cell/ or (human or humans or man or men or wom?n).ti.)

49. 47 not 48

50. 36 and 49

**Science Citation Index - Expanded (web of science) and Conference proceedings**

Searched May 25^th^, 2020 Records identified: 998

Updated search April 13^th^, 2021 Records identified: 1050

#17 (#16 AND #15)

#16 TS=(random* OR control* OR RCT OR placebo OR group* OR trial*)

#15 (#14 AND #3)

#14 (#13 OR #12 OR #11 OR #10 OR #9 OR #8 OR #7 OR #6 OR #5 OR #4)

#13 TS=(pulmonary edema)

#12 TS=(acute kidney failure or acute renal injuries)

#11 TS=(acute hepatic failure or fulminating hepatic failure or renal failure or acute tubular necrosis)

#10 TS=(sepsis or septic shock)

#9 TS=(trauma and (ICU* or intensive care))

#8 TS=(severe trauma or multiple trauma)

#7 TS=(respiratory distress syndrome or ARDS or respiratory failure)

#6 TS=(acute lung injury or respiratory failure)

#5 TS=(shock)

#4 TS=(critically ill or acutely ill or intensive care or critical care or ICU*)

#3 #2 OR #1

#2 TS=((fluid overload or hyperhydration or overhydration or positive fluid balance or hydration* or edema or water electrolyte imbalance) and (furosemide or torsemide or bumetanide or ethacrynic acid or azosemide or diuretic*))

#1 TI=(furosemide or torsemide or bumetanide or ethacrynic acid or azosemide or diuretic*)

**BIOSIS Previews (web of science)**

Searched May 25^th^, 2020 Records identified: 752

Updated search April 13^th^, 2021 Records identified: 790

#17 (#16 AND #15)

#16 TS=(random* OR control* OR RCT OR placebo OR group* OR trial*)

#15 (#14 AND #3)

#14 (#13 OR #12 OR #11 OR #10 OR #9 OR #8 OR #7 OR #6 OR #5 OR #4)

#13 TS=(pulmonary edema)

#12 TS=(acute kidney failure or acute renal injuries)

#11 TS=(acute hepatic failure or fulminating hepatic failure or renal failure or acute tubular necrosis)

#10 TS=(sepsis or septic shock)

#9 TS=(trauma and (ICU* or intensive care))

#8 TS=(severe trauma or multiple trauma)

#7 TS=(respiratory distress syndrome or ARDS or respiratory failure)

#6 TS=(acute lung injury or respiratory failure)

#5 TS=(shock)

#4 TS=(critically ill or acutely ill or intensive care or critical care or ICU*)

#3 #2 OR #1

#2 TS=((fluid overload or hyperhydration or overhydration or positive fluid balance or hydration* or edema or water electrolyte imbalance) and (furosemide or torsemide or bumetanide or ethacrynic acid or azosemide or diuretic*))

#1 TI=(furosemide or torsemide or bumetanide or ethacrynic acid or azosemide or diuretic*)

**Latin American Caribbean Health Sciences Literature (LILACS)**

Searched May 25^th^, 2020 Records identified: 264

Updated search April 13^th^, 2021 Records identified: 284

(tw:((fluid overload OR hyperhydration OR overhydration OR positive fluid balance OR hydration OR water electrolyte imbalance OR furosemide OR torsemide OR bumetanide OR ethacrynic acid OR azosemide OR diuretic))) AND (tw:((critically ill OR acutely ill OR intensive care OR critical care OR icu))) AND (tw:((randomized OR randomised OR random OR randomly OR control OR controlled OR rct OR placebo OR group OR trial)))

**Similar search strategy is applied to the following 4 Chinese databases:**

**China National Knowledge Infrastructure (CNKI)**

Searched June 3^rd^, 2020 Records identified: 118

Updated search April 29^th^, 2021 Records identified: 130

**Wanfang database**

Searched June 3^rd^, 2020 Records identified: 203

Updated search April 29^th^, 2021 Records identified: 243

**VIP** **Chinese Science Journals Database**

Searched June 3^rd^, 2020 Records identified: 226

Updated search April 29^th^, 2021 Records identified: 244

**Sinomed**

Searched June 3^rd^, 2020 Records identified: 195

Updated search April 29^th^, 2021 Records identified: 226

**Search through other resources:**

ClinicalTrials.gov, WHO International Clinical Trials Registry Platform (ICTRP), EU Clinical Trial Register, Australian New Zealand Clinical Trials Registry (ANZCTR), US Food and Drug Administration (FDA), European Medicines Agency (EMA), and Google scholar have been search without finding new relevant studies.

# **S3. Post hoc subgroup analysis for the comparison of loop diuretics vs placebo/no intervention**

The comparison loop diuretics vs placebo/no intervention consists of six trials. The control group in this comparison is placebo, no diuretics, or standard of care. Four out of six trials reported use of diuretics in the control group. To assess if use of diuretics in the control group could influence the results, we made post hoc subgroup analyses for the comparison with the following two subgroups:

- Diuretics is reported administered in the control group [37, 52, 54-55]
- Diuretics is not reported administered in the control group [56-57]

**Table S2.** Administration of loop diuretics in the control group in the comparison loop diuretics vs placebo/no intervention

| **Loop diuretic vs placebo/no intervention** | | |
| --- | --- | --- |
| **Trial** | **Intervention group** | **Control group** |
| Bagshaw [52] | Furosemide | **Placebo**  The cumulative dose of furosemide for the intervention group was not reported. Protocol violations with supplementary diuretic therapy was reported as 76 events in 11 out of 36 patients in the placebo group. Det dose of supplemental diuretics were not reported. |
| Berthelsen [37] | Furosemide | **Standard of care**  The cumulative furosemide dose was reported as mg/kg. Median dose in the group with loop diuretics: 9.0 (4.6 - 14.5) and median dose in standard of care: 2.0 (0.0 – 13.0). |
| Cardoso [55] | Furosemide | **Standard of care**  The mean dose of furosemide in the intervention group was 78.3 (29.5) mg/day and 44.8 (23.6) mg/day in control group. |
| Cinotti [54] | Furosemide | **No diuretics**  No diuretics was allowed in the control group but could be administered as rescue therapy in case of acute pulmonary oedema or de novo heart failure.  The cumulative dose of furosemide in the intervention group was 160 mg (80-285) and in the control group 100 mg (40-160). |
| Hamishehkar [56] | Furosemide | **No diuretics**  The cumulative dose of furosemide in the intervention group was not reported. No report of administration of diuretics in control group. |
| Sanchez [57] | Torsemide | **No diuretics**  The cumulative dose of torsemide was not reported for the intervention group. No diuretics was reported in the control group. |

# **S4. Detailed characteristics of included trials**

**Table S3.** Detailed characteristics of the included trials

| **Bagshaw et al. 2017** [52] | |  |
| --- | --- | --- |
| **Methods** | Multicentre, blinded, randomised clinical trial |  |
| **Participants** | **Sample size:** n=73 randomised (experimental: 37, control: 36) 72 analysed for outcomes.  **Sex (M/F):** 57/16  **Age (mean):** 64  **Country:** Canada/Australia  **Setting:** AKI patients in mixed ICUs.  **Inclusion criteria:** 1) evidence of early AKI (RIFLE category – RISK); 2) peripheral or central intravenous catheter and urinary catheter; 3) ≥ 2 criteria for the systemic inflammatory response syndrome within 24 hours of screening and 4) achieved immediate resuscitation goals based on judgement of the treating physician and including one or more of the following: fluid resuscitation and/or vasoactive therapy to achieve mean arterial pressure ≥ 65 mmHg, central venous pressure ≥ 8 cm H_2_O, central venous oxygen saturation ≥ 70% (if measured) and or cardiac index ≥ 2.5 L/min/1.73 m^2^ (if measured)  **Exclusion criteria:** 1) age < 18 years; 2) confirmed or suspected pregnancy; 3) suspected or confirmed obstructive ethology for AKI; 4) ≥ stage 4 chronic kidney disease, end stage kidney disease receiving maintenance dialysis or kidney transplantation; 5) recent RRT during ICU or index hospitalisation; 6) recovering AKI defined as a ≥ 25% or 44.2 µmol/L decline from peak increase in serum creatinine; 7) acute pulmonary oedema mandating urgent furosemide administration or RRT initiation or patient was already receiving a continuous furosemide infusion; 8) moribund status with expected death within 24 hours or significant limitations of medical therapy; 9) suspected or known allergy to furosemide; and 10) prior enrolment. |  |
| **Interventions** | **Experimental:** Furosemide bolus of 0.4mg/kg followed by a continuous infusion of furosemide with a starting dose of 0.05 mg/kg/h. Goal directed titration. Max. 0.4 mg/kg/h.  **Control:** placebo (saline)  **Co-intervention:** none  **Duration:** minimum 24 hours; maximum 7 days |  |
| **Outcomes** | **Primary outcome:** worsening of AKI, defined as progression from RIFLE category – RISK to a more severe category of AKI (INJURY, FAILURE or receipt of RRT) in the 7 days following randomisation.  **Secondary outcomes:** differences in cumulative fluid balance, serum electrolytes, acid-base status, rate of RRT initiation, rates of renal recovery, and hospital mortality between furosemide and placebo groups, respectively. |  |
| **Notes** | 1 patient in the control group did not receive the intervention and was not included in the analysis.  The trial was terminated early after 72 participants (216 planned participants) due to low recruitment, limited funding, influenza pandemic in 2009, and shortage of furosemide in North America in 2011.  Author contacted in January 2021 and response received in February 2021 with clarifications to data. Adverse events and reactions were not divided in serious and not serious.  Extra data on electrolytes were received. The data was not in a format that could be used in a meta-analysis. |  |
| **Berthelsen et al. 2018** [37] | |  |
| **Methods** | Multicentre, unblinded, randomised clinical trial |  |
| **Participants** | **Sample size:** 23 randomised (20 analysed)  **Sex (M/F):** 12:8  **Age (mean):** 72  **Country:** Denmark  **Setting:** AKI patients in two mixed ICUs.  **Inclusion criteria:** 1) age 18 years or older; 2) AKI defined according to the Kidney Disease Improving Global Outcomes (KDIGO) criteria; 3) renal recovery score (RS) ≤60%; 4) fluid overload defined as a positive fluid balance of at least 10% of ideal body weight; 5) able to undergo randomisation within 12 hours of fulfilling the other inclusion criteria.  **Exclusion criteria:** 1) known allergy to furosemide or sulphonamides; 2) known prehospitalisation advanced chronic kidney disease (eGFR < 30 mL/min/1.73m^2^ or chronic renal replacement therapy); 3) severe hypoxic respiratory failure (FiO_2_ > 80% and positive end-expiratory pressure (PEEP) > 10 cm H_2_O); 4) severe burn injury (≥ 10% total burned surface area); 5) severe dysnatraemia (plasma concentration < 120 or > 155 mmol/L); 6) hepatic coma; 7) mentally disabled undergoing forced treatment; 8) pregnancy/breastfeeding; 9) lack of commitment for ongoing life support including renal replacement therapy (RRT); and 10) lack of informed consent. |  |
| **Interventions** | **Experimental:** fluid removal to achieve a negative fluid balance of ≥ 1mL/kg/hour. First choice was furosemide infusion, and if it was insufficient according to goal assessed after 8 hours the patient was changed to continuous RRT to achieve the goal.  **Control:** standard of care.  **Co-intervention:** none  **Duration:** 5 days |  |
| **Outcomes** | **Primary outcome:** cumulative fluid balance 5 days after randomisation.  **Secondary outcomes:** 1) mean daily fluid balance during ICU stay; 2) cumulative fluid balance during the entire ICU stay; 3) time to neutral cumulative fluid balance; 4) number of patients with one or more major protocol violations; 5) accumulated SARs in each intervention arm during the ICU stay.  **Exploratory outcomes:** 1) all-cause mortality at day 90; 2) Days alive and out of hospital within 90 days of follow-up; 3) days alive without mechanical ventilation within 90 days of follow-up; 4) Days alive without vasopressor/inotropic therapy within 90 days follow-up; 5) days alive without RRT within 90 days follow-up; 6) renal recovery at day 90. |  |
| **Notes** | The inclusion criteria were changes after inclusion of the first two participants. Three patients did not receive the allocated treatment and was not included in the analysis.  The trial was terminated early due to futility. Less than half of the planned sample size was included.  Author contacted in January 2021 and clarifying information and all raw data were provided. From raw data we could extract data not presented in the article for several of our outcomes (length of stay in ICU, creatinine, electrolytes, resolution of fluid overload, SAEs). The data of creatinine and electrolytes were screwed and not suitable for meta-analysis. |  |
| **Brown et al. 2019** [53] | |  |
| **Methods** | Single centre, randomised clinical trial |  |
| **Participants** | **Sample size:** 26 randomised (25 analysed)  **Sex (M/F):** 15/10  **Age (median):** 55  **Country:** Australia  **Setting:** Mixed ICU  **Inclusion criteria:** 1) age above 18 years; 2) physician decision to administer an intravenous diuretic; 3) anticipated length of stay for more than 24 hours; 4) existing intra-arterial or central venous catheter and urinary catheter.  **Exclusion criteria:** 1) allergy to furosemide or acetazolamide or other sulphonamides; 2) end-stage renal failure; 3) long-standing use of diuretics: 4) dose of any diuretic in the preceding 12 hours; 5) significant acid-base disturbance at the time of enrolment (pH < 7.3 or > 7.5); and 6) treatment with RRT. |  |
| **Interventions** | **Experimental:** single bolus of 40 mg furosemide  **Control:** single bolus of 500 mg acetazolamide  **Co-intervention:** none  **Duration:** 6 hours |  |
| **Outcomes** | 1) Change in cumulative fluid balance 6 hours after the intervention.  2) Change in the cumulative urine output and serum and urine biochemistry for 6 hours before and 6 hours after the intervention. |  |
| **Notes** | One patient withdrew consent and was not included in the analysis.  Author contacted in February and response received. |  |
| **Cardoso et al. 2013** [55] | |  |
| **Methods** | Single centre, single blinded, randomised clinical trial |  |
| **Participants** | **Sample size**: n=72 (experimental: 34, control: 38 - randomised and analysed)  **Sex (M/F):** 59/13  **Age (mean):** 58  **Country:** Brazil  **Setting:** Patients with decompensated heart failure in Medical ICU  **Inclusion criteria:** 1) ≥ 18 years of age; 2) NYHA class IV; 3) ejection fraction < 45%; 4) decompensated heart failure; 5) and presence of two or more signs of water retention.  **Exclusion criteria:** 1) serum urea > 150 mg/dL; 2) serum creatinine level > 3 mg/dL; 3) peritoneal dialysis; 4) haemodialysis; 5) severe aortic stenosis; 6) and insulin dependent diabetes mellitus. |  |
| **Interventions** | **Experimental:** furosemide 120 mg/day as starting dose - titrated according to an algorithm.  **Control:** standard of care  **Co-intervention:** none described  **Duration:** unclear. All patients were followed until free from congestion |  |
| **Outcomes** | **Primary outcome:** Time to being free from congestion  **Secondary outcome:** worsening of renal function |  |
| **Notes** | Author contacted January and March 2021 without response |  |
| **Cinotti et al. 2021** [54] | |  |
| **Methods** | Multicentre, single blinded, randomised clinical trial. |  |
| **Participants** | **Sample size:** 171 randomised (166 analysed)  **Sex (M/F):** 122/44  **Age (mean):** 66  **Country:** France  **Setting:** Mixed ICU  **Inclusion criteria:** 1) ≥ 18 years old; 2) admitted to an ICU and receiving invasive mechanical ventilation (FiO_2_ ≤ 60%, and PEEP ≤ 10 cm H_2_O on inclusion); 3) positive fluid balance defined as in-ICU weight increase ≥ 3%; 4) haemodynamic stable (no vasoactive drugs).  **Exclusion criteria:** 1) pregnancy; 2) withdrawal of life-sustaining therapies in the 24 hours after admission; 3) allergy to furosemide; 4) admission for decompensated cirrhosis; central neurologic injury, and chronic kidney failure; 5) when treatment with diuretics are mandatory (acute pulmonary oedema, heart failure with a reduced ejection fraction ≤ 30%). |  |
| **Interventions** | **Experimental:** furosemide once or twice a day until successful extubation. Dose adjusted to every patient.  **Control:** diuretic administration prohibited  **Co-intervention:** diuretics is used as rescue therapy in case of pulmonary oedema or de novo heart failure.  **Duration:** until successful extubation. |  |
| **Outcomes** | **Primary outcome:** fluid balance defined as weight variation from weight on randomisation to weight on successful extubation.  **Secondary outcomes:** 1) rate of extubation failure; 2) duration of mechanical ventilation from randomisation to successful weaning; 3) number of ventilatory free days by day 28; 4) length of stay in ICU; 5) ICU mortality; 6) 60-day mortality post randomisation. |  |
| **Notes** | After inclusion of 36 patients the inclusion criteria were modified, which might have changed the study population to some degree. 5 patients were excluded in the intervention group because of violation of an inclusion criterion.  Authors were contacted and response received. |  |
| **Hamishehkar et al. 2017** [56] | |  |
| **Methods** | Multicentre, randomised clinical trial |  |
| **Participants** | **Sample size:** n=106 randomised (analysed: experimental: 50, control: 50)  **Sex (M/F):** 64/36  **Age (mean):** 63  **Country:** Iran  **Setting:** patients with AKI in two surgical ICUs.  **Inclusion criteria:** patients with increase in creatinine to more than 150% - 300% or urine output decreased to < 0.5 cc/kg/hour for 12 hours or more.  **Exclusion criteria:** 1) previous history of AKI; 2) RRT; 3) renal transplantation; 4) urinary system obstruction; 5) previous history of diuretic use; 6) alkalosis, or 7) hypovolemia. |  |
| **Interventions** | **Experimental:** 40-80 mg of furosemide iv followed by infusion of 1-5 mg/hour according to urine output.  **Control:** no diuretics  **Co-intervention:** RRT was started in case of fluid overload resistant to medical therapy, severe acidosis resistant to medical treatment, severe electrolyte imbalance resistant to treatment, uremic signs or symptoms, and progressive azotaemia in the absence of uraemia.  **Duration:** 7 days |  |
| **Outcomes** | Evaluate biomarkers (blood urea nitrogen, creatinine, plasma neutrophil gelatinase-associated lipocalin (NGAL), urine NGAL) in AKI patients. |  |
| **Notes** | 106 patients were randomised but only 100 patients were analysed. It is unclear to which group the 6 excluded patients belonged. Reasons of exclusion: two patients died, three were ineligible after enrolment, and one declined to participate.  The authors were contacted March and April 2021 without response. |  |
| **Han 2019** [59] | |  |
| **Methods** | Single centre, blinded, randomised clinical trial |  |
| **Participants** | **Sample size:** n=248 (experimental:124, control: 124) randomised and analysed  **Sex (M/F):** 87:161  **Age (mean):** 45  **Country:** China  **Setting:** Cardiac intensive care patients  **Inclusion criteria:** 15-65 years of age with signs of fluid overload  **Exclusion criteria:** 1) age below 15 or above 65 years; 2) cardiac issues (systolic blood pressure < 80 mmHg) and renal instability (serum creatinine > 3.99 mg dL^-1^); 3) lack of informed consent; 4) pregnancy and breastfeeding; 5) patients who needed dialysis or ultrafiltration on the time of enrolment. |  |
| **Interventions** | **Experimental:** infusion of furosemide: 0.8 mg/kg/hour  **Control:** infusion of ethacrynic acid 0.5 mg/kg/hour  **Co-intervention:** none  **Duration:** maximum 3 days |  |
| **Outcomes** | **Primary outcome:** increase urine output in young and adult patients with fluid overload.  **Secondary outcome:** compare the efficacy and safety of furosemide with ethacrynic acid.  The primary and secondary outcomes were different between protocol and article. These outcomes are from the article. |  |
| **Notes** | Author contacted February and March 2021 without response. |  |
| **Ng et al. 2020** [60] | |  |
| **Methods** | Single centre, open label, randomised clinical trial |  |
| **Participants** | **Sample size:** n=33 randomised and analysed (experimental: 15, control: 18)  **Sex (M/F):** 25/8  **Age (mean):** 56  **Country:** USA  **Setting:** Patients with acute heart failure admitted to an ICU.  **Inclusion criteria:** 1) Acute heart failure with signs or symptoms of volume overload 2) Serum sodium < 135 mEq/L at time of or within first 48 hours of hospitalization 3) Informed consent.  **Exclusion criteria: 1)** Severe symptomatic hyponatremia requiring acute treatment, 2) moderate to severe lever impairment, 3) Severe renal impairment upon admission (creatinine clearance < 20 mL/min), 4) Renal replacement therapy dependent or required upon admission, 5) Acute coronary syndrome on admission, 6) Evidence of cardiogenic shock or requiring intravenous vasopressors, 7) Pregnancy, 8) Concomitant use of strong CYP3A4 inhibitors (clarithromycin, ketoconazole, itraconazole, ritonavir, indinavir, nelfinavir, saquinavir, nefazodone, and telithromycin) |  |
| **Interventions** | **Experimental:** Furosemide infusion of 5 mg/hour with the option to titrate to a maximum of 20 mg/hour after the first 24 hours.  **Control:** Tablet tolvaptan 30 mg daily with the option to titrate up to a maximum of 60 mg daily after the first 24 hours.  **Co-intervention:** Baseline thiazide diuretics were discontinued during the trial. In case of maximum tolvaptan or furosemide administration metolazone could be added in both groups to achieve the desired urine output of 100 mL/hour.  **Duration:** maximum 96 hours (4 days) |  |
| **Outcomes** | **Primary outcome:** 1) Mean urine output at 24 hours post randomisation 2) Mean change in serum creatinine at 24 hours post randomisation.  **Secondary outcomes:** 1) Urine output and sodium change at 8, 48, 96 hours post-randomisation 2) Proportion of patients requiring escalation of study drug dose or the addition of metolazone 3) Change in self-rated dyspnoea (Likert Scale) at 24 and 96 hours 4) change in estimated glomerular filtration rate at 24, 48, and 96 h post randomisation 5) Incidence of acute increases in serum creatinine ≥26.5 µmol/L (0.3 mg/dL), 6) In-hospital mortality, 7) changes in biomarkers (plasma renin activity, copeptin, plasma N-terminal pro b-type natriuretic peptide, cystatin C, and Urinary neutrophil gelatinase-associated lipocalin. |  |
| **Notes** | Inclusion and exclusion criteria and outcomes in protocol and article differed. Data were only registered as long patients followed the protocol. The sample size was small, and many participants were discontinued from the study protocol after 48 hours because of clinical resolution or protocol violation according to diuretics (switch to bumetanide), so the results beyond 48 hours should be interpreted with caution. In-hospital mortality was not reported in article, but data can be found on Clincal.Trial.gov. No SAE/AE was reported on Clincal.Trial.gov.  First sample size calculation was on 50 participants. Due to slow enrolment re-calculation of sample size was performed and sample size revised to 34-46 subjects. 33 participants included. Only per protocol analysis were performed.  Authors were contacted in April 2021 and answer received. |  |
| **Sanchez et al. 2003** [57] | |  |
| **Methods** | Randomised clinical trial |  |
| **Participants** | **Sample size:** n=40 randomised and analysed (experimental: 20, control: 20)  **Sex (M/F):** no info  **Age (mean):** 68  **Country:** Spain  **Setting:** patients with AKI in ICU.  **Inclusion criteria:** 1) diuresis < 1 ml/kg/hour; 2) creatinine clearance < 60 ml/min; 3) hemodynamic resuscitation (mean arterial pressure ≥70 mmHg, CVP ≥12 mmHg).  **Exclusion criteria:** not described. |  |
| **Interventions** | **Experimental:** torsemide – dose not described  **Control:** no intervention  **Co-intervention:** RRT on indication  **Duration:** maximum 7 days |  |
| **Outcomes** | The effect of low dose dopamine and torsemide on creatinine clearance and the need for RRT in critically ill septic oliguric patients. | |
| **Notes** | Only an abstract available.  Participants were randomised to 4 groups: control, dopamine < 3mg/kg/min, torsemide iv bolus, and torsemide and dopamine. We only extracted data from the torsemide and control group. We interpreted the control group as no diuretics. No data on diuretics in the control group was reported.  No contact information was found on the authors. | |
| **Wappler et al. 1991** [58] | |  |
| **Methods** | Single centre, randomised clinical trial | |
| **Participants** | **Sample size:** n=12 randomised and analysed (experimental: 6, control: 6)  **Sex (M/F):** 5/7  **Age (mean):** 68  **Country:** Germany  **Setting:** postoperative patients in a cardio surgical intensive care unit  **Inclusion criteria:** not described  **Exclusion criteria:** not described | |
| **Interventions** | **Experimental:** bolus of 40 mg furosemide followed by infusion of 20 mg/hour. In case of decrease in diuresis bolus of 40 mg furosemide was allowed.  **Control:** bolus of 12 mg piretanide followed by infusion of 6 mg/hour. In case of decrease in diuresis bolus of 12 mg piretanide was allowed.  **Co-intervention:** all patients received 100 mg of spironolactone every 8 hours. Mannitol infusion was allowed.  **Duration:** 40 hours | |
| **Outcomes** | Comparison of the effect of furosemide and piretanide in patients in cardiosurgical intensive care unit. | |
| **Notes** | No contact information on the authors were found. | |

# **S5. Overall risk of bias for all included trials**

We used ROB2 tool in assessing the risk of bias. Trials or outcomes were judged at overall low risk of bias if all five domains had low risk of bias. Trials or outcomes were judged at overall high risk of bias when some concerns or high risk of bias was judged in one or more domains.

**Table S4.** Overall risk of bias assessment for all included trials

| **Study** | **Rando-misation** | **Deviations from intervention** | **Missing outcome data** | **Measure-ment of the outcome** | **Selection of the reported results** | **Overall risk of bias** |
| --- | --- | --- | --- | --- | --- | --- |
| Bagshaw | Low | Low | Low | Low | **Some concerns^1^** | **High^2^** |
| Berthelsen | Low | **High^3^** | **Some concerns^4^** | Low | Low | **High^5^** |
| Brown | Low | Low | Low | Low | **Some concerns^6^** | **High** |
| Cardoso | **Some concerns^7^** | **High^8^** | **High^9^** | **High^10^** | **Some concerns^11^** | **High** |
| Cinotti | Low | **Some concerns^12^** | Low | **Some concerns^13^** | **Some concerns^14^** | **High** |
| Hamishehkar | Low | **High^15^** | **High^16^** | Low | **Some concerns^17^** | **High** |
| Han | **Some concerns^18^** | Low | Low | Low | **Some concerns^19^** | **High** |
| Ng | **Some concerns^20^** | **High^21^** | Low | **High^22^** | **Some concerns^23^** | **High** |
| Sanchez | **Some concerns^24^** | **High^25^** | **High^26^** | **Some concerns^27^** | **Some concerns^28^** | **High** |
| Wappler | **Some concerns^29^** | **High^30^** | **Some concerns^31^** | Low | **Some concerns^32^** | **High** |

1. Full trial protocol and statistical analysis plan were not available, but the trial was registered on ClincalTrials.gov. SAE/AE were not outcomes but reported.
2. The calculated sample size was 216 participants, but the trial was stopped after 73 included participants.
3. The trial was not blinded which might have affected the treatment in the standard of care group. Inclusion criteria were changed during the trial this might have changed the patient population to some degree.
4. Three participants (13%) were excluded out of 23 randomised participants. Data of the excluded participants were not used in the analyses. No sensitivity analyses could be performed due to small sample size.
5. The trial was stopped prematurely with less than half of the planned participants included.
6. No protocol or analysis plan is available, but the trial was registered at New Zealand Clinical Trials Registry. In the trial registry 15 secondary outcomes were listed, but only 8 outcomes were reported in the article.
7. Randomisation process was not described.
8. Only the participants were blinded for the intervention. The control group was standard of care. The knowledge of the allocation group might have affected the treatment in the standard of care group due to the Hawthorne effect. No information reported on exclusions, withdrawal and lost to follow-up.
9. No information about missing data. No CONSORT-diagram reported.
10. Outcome assessors were not blinded. The primary outcome “being free from congestion” were not defined. The lack of blinding has the potential to affect the assessment “being free from congestion” which is partly a subjective outcome. Objective outcomes are less or not affected by the lack of blinding.
11. No protocol, statistical analysis plan or trial registry available.
12. Change in inclusion criteria during the trial, which changed the randomised patient population to some degree. Five participants were excluded from the furosemide group due to inadequate inclusion criteria post randomisation. It is not reported if these five participants had received the intervention. They were excluded from the intention-to-treat analysis. Single-blinded trial.
13. The Primary outcome was fluid balance at the time of extubation. Knowledge of the intervention group might affect when the patients are judged ready to extubate. Other objective outcomes are less affected by the lack of blinding.
14. No protocol or statistical plan available, but the trial was registered on ClincalTrials.gov.
15. Discrepancies between trial registry and article. In the trial registry the trial was stated as double blinded, but blinding was not described in the article. The control group was reported to receive placebo in the trial registry, but in the article the control group was described as standard treatment with no diuretics. Six participants are excluded for the intention-to-treat analysis (two died during the trial, three were ineligible after enrolment and one declined to participate). Allocation groups not revealed for the excluded participants.
16. Two participants who died during the trial were excluded from the analysis and not included in the reported ICU mortality. The allocation group is unknow and might have an impact.
17. No published protocol or statistical analysis plan. The trial was registered at Iranian Registry of Clinical Trials.
18. Randomisation process not described.
19. Short protocol without statistical analysis plan was attached in the trial registry. One secondary outcome in the protocol was not reported in the article.
20. Allocation process not described.
21. No blinding. Only data on participants who remained on study protocol were registered. After the first 48 hours the trial intervention was stopped in a large percentage of participants - mainly in the tolvaptan group due to clinical decisions (clinical resolution or diuretic switch to bumetanide) and additional medicine as metolazone was used more in the tolvaptan group compared to the furosemide group. This especially affected the outcomes from 48 hours to 96 hours. Per protocol analyses were performed.
22. Due to no blinding the clinicians might be more prone to remove participants from the study protocol if they find other treatment options more advantageous. Only seven participants remained in protocol until 96 hours in the tolvaptan group and 11 patients in the furosemide group. The attrition from study protocol was due to clinical resolution or switch to bumetanide.
23. Three outcomes in the protocol were not reported in the article – some degree of selective reporting cannot be ruled out. SAE were only reported on ClincalTrials.gov – not in the article.
24. Randomisation and allocation process not reported.
25. Blinding and analysis methods were not described.
26. Unclear how many participants were randomised, and if all randomised participants were included in the analyses. No information on withdrawal, lost to follow-up and missing data.
27. Renal replacement therapy was stated to be initiated according to predefined criteria, but these criteria are not reported. No blinding.
28. Only published as an abstract. No protocol, statistical analysis plan or trial registry could be found.
29. Randomisation process not described.
30. No information about blinding or deviations for intended interventions.
31. Unclear how many participants were randomised and if all randomised participants were analysed.
32. No published protocol, statistical analysis plan or registration in a trial registry.

# **S6. Comparison: loop diuretics vs placebo/no intervention**

## **S6a. Risk of bias of all outcomes**

We used the ROB2 tool when assessing the risk of bias. Outcomes were judged at overall low risk of bias if all five domains had low risk of bias. Outcomes were judged at overall high risk of bias when some concerns or high risk of bias was judged in one or more domains.

**Table S5.** Risk of bias assessment on all-cause mortality for loop diuretics vs. placebo/no intervention

| **Study** | **Rando-misation** | **Deviations from intervention** | **Missing outcome data** | **Measure-ment of the outcome** | **Selection of the reported results** | **Overall risk of bias** |
| --- | --- | --- | --- | --- | --- | --- |
| Bagshaw | Low | Low | Low | Low | **Some concerns^1^** | **High^2^** |
| Berthelsen | Low | **High^3^** | **Some concerns^4^** | Low | Low | **High^5^** |
| Cinotti | Low | **Some concerns^6^** | Low | Low | **Some concerns^7^** | **High** |
| Hamishehkar | Low | **High^8^** | **High^9^** | Low | **Some concerns^10^** | **High** |

1. Full trial protocol and statistical analysis plan were not available, but the trial was registered on ClincalTrials.gov.
2. The calculated sample size was 216 participants, but the trial was stopped after 73 included participants.
3. The trial was not blinded which might have affected the treatment in the standard of care group. Inclusion criteria were changed during the trial this might have changed the patient population to some degree.
4. Three participants (13%) were excluded out of 23 randomised participants. Data of the excluded participants were not used in the analyses. No sensitivity analyses could be performed due to small sample size.
5. The trial was stopped prematurely with less than half of the planned participants included.
6. Change in inclusion criteria during the trial, which changed the patient population to some degree. Five participants were excluded from the furosemide group due to inadequate inclusion criteria post randomisation. It is not reported if these five participants had received the intervention. They were excluded from the intention-to-treat analysis. Single-blinded trial.
7. No protocol or statistical plan available, but the trial was registered on ClincalTrials.gov.
8. Discrepancies between trial registry and article. In the trial registry the trial was stated as double blinded, but blinding was not described in the article. The control group was reported to receive placebo in the trial registry, but in the article the control group was described as standard treatment with no diuretics. Six participants are excluded for the intention-to-treat analysis (two died during the trial, three were ineligible after enrolment and one declined to participate). Allocation groups not revealed for the excluded participants.
9. Two participants who died during the trial were excluded from the analysis and not included in the reported ICU mortality. The allocation group is unknow and might have an impact.
10. No published protocol or statistical analysis plan. The trial was registered at Iranian Registry of Clinical Trials.

**Table S6.** Risk of bias assessment on serious adverse events for loop diuretics vs. placebo/no intervention

| **Study** | **Rando-misation** | **Deviations from intervention** | **Missing outcome data** | **Measure-ment of the outcome** | **Selection of the reported results** | **Overall risk of bias** |
| --- | --- | --- | --- | --- | --- | --- |
| Bagshaw | Low | Low | Low | Low | **Some concerns^1^** | **High^2^** |
| Berthelsen | Low | **High^3^** | **Some concerns^4^** | Low | Low | **High^5^** |
| Cardoso | **Some concerns^6^** | **High^7^** | **High^8^** | Low | **Some concerns^9^** | **High** |
| Cinotti | Low | **Some concerns^10^** | Low | Low | **Some concerns^11^** | **High** |
| Hamishehkar | Low | **High^12^** | **High^13^** | Low | **Some concerns^14^** | **High** |
| Sanchez | **Some concerns^15^** | **High^16^** | **High^17^** | **Some concerns^18^** | **Some concerns^19^** | **High** |

1. Full trial protocol and statistical analysis plan were not available, but the trial was registered on ClincalTrials.gov. SAE/AE were not outcomes but reported.
2. The calculated sample size was 216 participants, and the trial was stopped after 73 included participants.
3. The trial was not blinded which might have affected the treatment in the standard of care group. Inclusion criteria were changed during the trial, this might have affected the patient population.
4. Three participants (13%) were excluded out of 23 randomised participants. Data of the excluded participants were not used in the analyses. No sensitivity analyses could be performed due to small sample size.
5. The trial was stopped prematurely with less than half of the planned participants included.
6. Randomisation process was not described.
7. Only the participants were blinded for the intervention. The control group was standard of care. The knowledge of the allocation group might have affected the treatment in the standard of care group due to the Hawthorne effect. No information reported on exclusions, withdrawal and lost to follow-up.
8. No information about missing data. No CONSORT-diagram reported.
9. No protocol, statistical analysis plan or trial registry available.
10. Change in inclusion criteria during the trial which changed the patient population to some degree. Five participants were excluded from the furosemide group due to inadequate inclusion criteria post randomisation. It is not reported if these five participants had received the intervention. They were excluded from the intention-to-treat analysis. Single-blinded trial.
11. No protocol or statistical plan available, but the trial was registered on ClincalTrials.gov.
12. Discrepancies between trial registry and article. In the trial registry the trial was stated as double blinded, but blinding was not described in the article. The control group was reported to receive placebo in the trial registry, but in the article the control group was described as standard treatment with no diuretics. Six participants are excluded for the intention-to-treat analysis (two died during the trial, three were ineligible after enrolment and one declined to participate). Allocation groups not revealed for the excluded participants.
13. Six participants were excluded (5.7 %) from the intention-to-treat analysis (two died, one withdrew consent, and 3 were ineligible after enrolment). Allocation group not revealed. Missing data not mentioned in the trial.
14. No protocol or statistical plan available, but the trial was registered on Iranian Registry of Clinical Trials.
15. Randomisation and allocation process not reported.
16. Blinding not described.
17. Unclear how many participants were randomised, and if all randomised participants were included in the analyses. No information on lost to follow-up and missing data.
18. Renal replacement therapy was stated to be initiated according to predefined criteria, but these criteria are not reported. No blinding.
19. Only an abstract published. No protocol, statistical analysis plan or trial registry could be found.

**Table S7.** Risk of bias assessment on plasma creatinine for loop diuretics vs. placebo/no intervention

| **Study** | **Rando-misation** | **Deviations from intervention** | **Missing outcome data** | **Measure-ment of the outcome** | **Selection of the reported results** | **Overall risk of bias** |
| --- | --- | --- | --- | --- | --- | --- |
| Bagshaw | Low | Low | Low | Low | **Some concerns^1^** | **High^2^** |
| Berthelsen | Low | **High^3^** | **Some concerns^4^** | Low | **Some concerns^5^** | **High^6^** |
| Hamishehkar | Low | **High^7^** | **High^8^** | Low | **Some concerns^9^** | **High** |

1. No protocol or statistical analysis plan available, but the trial was registered at ClinicalTrials.gov.
2. The calculated sample size was 216 participants, and the trial was stopped after 73 included participants.
3. The trial was not blinded which might have affected the treatment in the standard of care group. Inclusion criteria were changed during the trial.
4. Three participants (13%) were excluded out of 23 randomised participants. Data of the excluded participants were not used in the analyses. No sensitivity analyses could be performed due to small sample size.
5. Plasma creatinine was not an outcome in this trial, but it was calculated by the review group from raw data delivered by the authors.
6. The trial was stopped prematurely with less than half of the planned participants included.
7. Discrepancies between trial registry and article. In the trial registry the trial was stated as double blinded, but blinding was not described in the article. The control group was reported to receive placebo in the trial registry, but in the article the control group was described as standard treatment with no diuretics. Six participants are excluded for the intention-to-treat analysis (two died during the trial, three were ineligible after enrolment and one declined to participate). Allocation groups not revealed for the excluded participants.
8. Six participants were excluded (5.7 %) from the intention-to-treat analysis (two died, one withdrew consent, and 3 were ineligible after enrolment). Allocation group not revealed. Missing data not mentioned in the trial.
9. No protocol or statistical plan available, but the trial was registered on Iranian Registry of Clinical Trials.

**Table S8.** Risk of bias assessment on proportion of participants without resolution of fluid overload for loop diuretics vs. placebo/no intervention

| **Study** | **Rando-misation** | **Deviations from intervention** | **Missing outcome data** | **Measure-ment of the outcome** | **Selection of the reported results** | **Overall risk of bias** |
| --- | --- | --- | --- | --- | --- | --- |
| Berthelsen | Low | **High^1^** | **Some concerns^2^** | Low | Low | **High^3^** |
| Cardoso | **Some concerns^4^** | **High^5^** | **High^6^** | **High^7^** | **Some concerns^8^** | **High** |

1. The trial was not blinded which might have affected the treatment in the standard of care group. Inclusion criteria were changed during the trial and the trial.
2. Three participants (13%) were excluded out of 23 randomised participants. Data of the excluded participants were not used in the analyses. No sensitivity analyses could be performed due to small sample size.
3. The trial was stopped prematurely with less than half of the planned participants included.
4. Randomisation process was not described. Single blinded trial.
5. Only the participants were blinded for the intervention. The control group was standard of care. The knowledge of the allocation group might have affected the treatment in the standard of care group due to the Hawthorne effect. No information reported on exclusions, withdrawal and lost to follow-up.
6. No information about missing data. No CONSORT-diagram reported.
7. Outcome assessors were not blinded and the primary outcome “being free from congestion” (resolution of fluid overload) were not defined. The lack of blinding has the potential to affect the assessment of this outcome.
8. No protocol, statistical analysis plan or trial registry available.

**Table S9**. Risk of bias assessment on number of days on mechanical ventilation for loop diuretics vs. placebo/no intervention

| **Study** | **Rando-misation** | **Deviations from intervention** | **Missing outcome data** | **Measure-ment of the outcome** | **Selection of the reported results** | **Overall risk of bias** |
| --- | --- | --- | --- | --- | --- | --- |
| Berthelsen | Low | **High^1^** | **Some concerns^2^** | Low | Low | **High^3^** |
| Cinotti | Low | **Some concerns^4^** | Low | **High^5^** | **Some concerns^6^** | **High** |

1. The trial was not blinded which might have affected the treatment in the standard of care group. Inclusion criteria were changed during the trial.
2. Three participants (13%) were excluded out of 23 randomised participants. Data of the excluded participants were not used in the analyses. No sensitivity analyses could be performed due to small sample size.
3. The trial was stopped prematurely with less than half of the planned participants included.
4. Change in inclusion criteria during the trial which changed the patient population to some degree. Five participants were excluded from the furosemide group due to inadequate inclusion criteria post randomisation. It is not reported if these five participants had received the intervention. They were excluded from the intention-to-treat analysis. Single-blinded trial.
5. The trial’s primary outcome was fluid balance on extubation. Knowledge of the intervention might have affected when the participants were assessed ready to extubate - even they had a described weaning protocol from mechanical ventilation in the article.
6. No protocol or statistical plan available, but the trial was registered on ClincalTrials.gov.

**Table S10.** Risk of bias assessment on length of stay for loop diuretics vs. placebo/no intervention

| **Study** | **Rando-misation** | **Deviations from intervention** | **Missing outcome data** | **Measure-ment of the outcome** | **Selection of the reported results** | **Overall risk of bias** |
| --- | --- | --- | --- | --- | --- | --- |
| Berthelsen | Low | **High^1^** | **Some concerns^2^** | Low | **Some concerns^3^** | **High^4^** |
| Cinotti | Low | **Some concerns^5^** | Low | Low | **Some concerns^6^** | **High** |

1. The trial was not blinded which might have affected the treatment in the standard of care group. Inclusion criteria were changed during the trial.
2. Three participants (13%) were excluded out of 23 randomised participants. Data of the excluded participants were not used in the analyses. No sensitivity analyses could be performed due to small sample size.
3. Length of stay was not an outcome in this trial, but it was calculated by the review group from raw data delivered by the authors.
4. The trial was stopped prematurely with less than half of the planned participants included.
5. Change in inclusion criteria during the trial which changed the patient population to some degree. Five participants were excluded from the furosemide group due to inadequate inclusion criteria post randomisation. It is not reported if these five participants had received the intervention. They were excluded from the intention-to-treat analysis. Single-blinded trial.
6. No protocol or statistical plan available, but the trial was registered on ClincalTrials.gov.

**Table S11.** Risk of bias assessment on adverse events not considered serious for loop diuretics vs. placebo/no intervention

| **Study** | **Rando-misation** | **Deviations from intervention** | **Missing outcome data** | **Measure-ment of the outcome** | **Selection of the reported results** | **Overall risk of bias** |
| --- | --- | --- | --- | --- | --- | --- |
| Bagshaw | Low | Low | Low | Low | **Some concerns^1^** | **High^2^** |
| Cinotti | Low | **Some concerns^3^** | Low | Low | **Some concerns^4^** | **High** |

1. No protocol or statistical analysis plan available, but the trial was registered at ClinicalTrials.gov.
2. The calculated sample size was 216 participants, and the trial was stopped after 73 included participants.
3. Change in inclusion criteria during the trial which changed the patient population to some degree.
4. No protocol or statistical plan available, but the trial was registered on ClincalTrials.gov.

**Table S12.** Risk of bias assessment on plasma sodium and potassium for loop diuretics vs. placebo/no intervention

| **Study** | **Rando-misation** | **Deviations from intervention** | **Missing outcome data** | **Measure-ment of the outcome** | **Selection of the reported results** | **Overall risk of bias** |
| --- | --- | --- | --- | --- | --- | --- |
| Bagshaw | Low | Low | Low | Low | **Some concerns^1^** | **High^2^** |
| Berthelsen | Low | **High^3^** | Low | Low | **Some concerns^4^** | **High^5^** |

1. No protocol or statistical analysis plan available, but the trial was registered at ClinicalTrials.gov.
2. The calculated sample size was 216 participants, and the trial was stopped after 73 included participants.
3. The trial was not blinded which might have affected the treatment in the standard of care group. Inclusion criteria were changed during the trial.
4. Plasma electrolytes were not an outcome in this trial, but it was calculated by the review group from raw data delivered by the authors.
5. The trial was stopped prematurely with less than half of the planned participants included.

**Table S13.** Risk of bias assessment on plasma chloride for loop diuretics vs. placebo/no intervention

| **Study** | **Rando-misation** | **Deviations from intervention** | **Missing outcome data** | **Measure-ment of the outcome** | **Selection of the reported results** | **Overall risk of bias** |
| --- | --- | --- | --- | --- | --- | --- |
| Berthelsen | Low | **High^1^** | Low | Low | **Some concerns^2^** | **High^3^** |

1. The trial was not blinded which might have affected the treatment in the standard of care group. Inclusion criteria were changed during the trial.
2. Plasma electrolytes were not an outcome in this trial, but it was calculated by the review group from raw data delivered by the authors.
3. The trial was stopped prematurely with less than half of the planned participants included.

## **S6b. Meta-analyses and TSA**

TSA was conducted for primary and secondary outcomes with meta-analysis.

**Fig. S1.** Meta-analysis and TSA for adverse events not considered serious for loop diuretics vs. placebo/no intervention

**
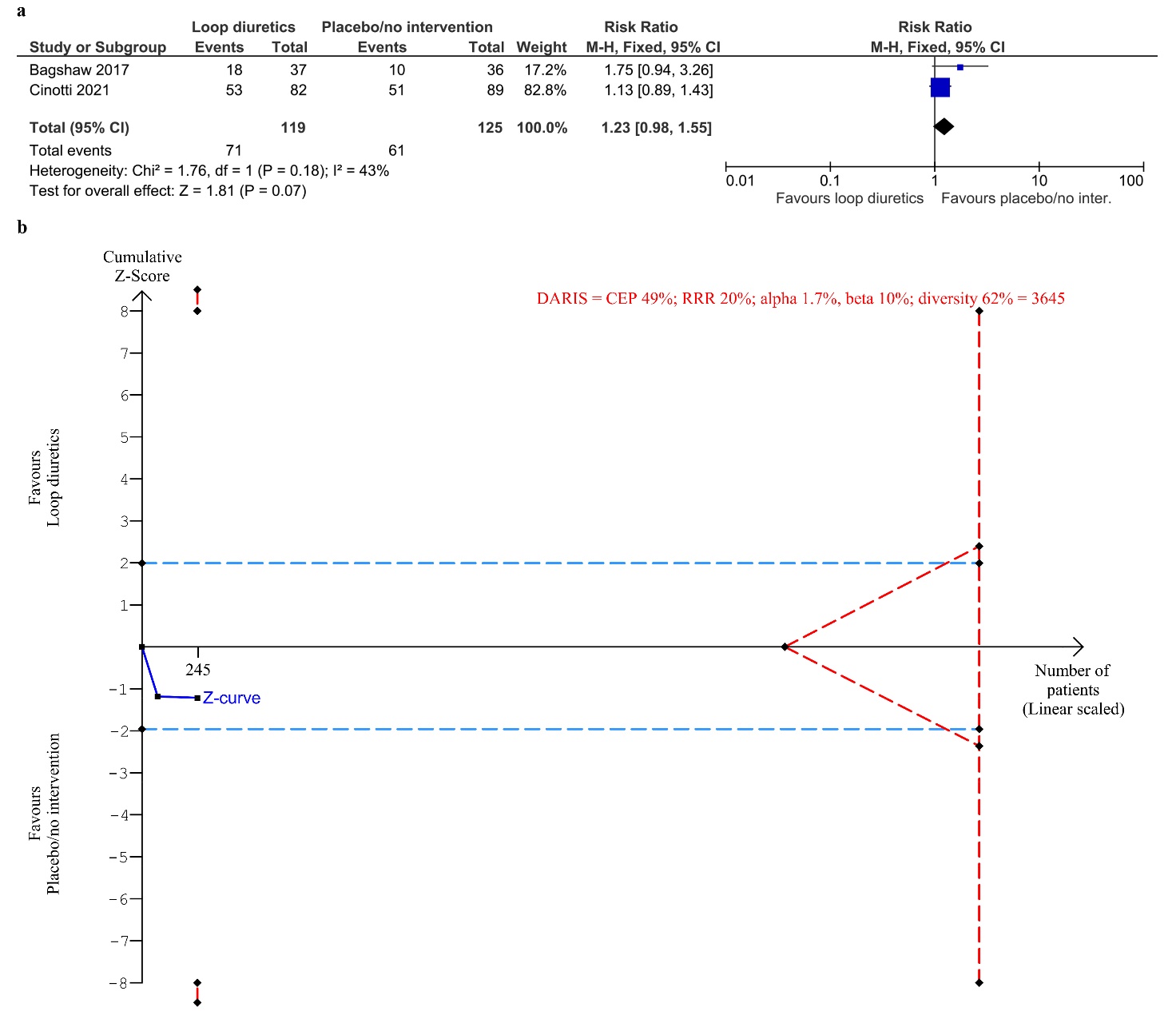
**

a) Meta-analysis. b) TSA. The diversity adjusted required information size (DARIS) was calculated according to the proportion of AE/ARs in the control group (CEP) of 49%; risk ratio reduction (RRR) of 20% in the experimental intervention group; alpha of 1.7%; beta of 10% (90% power); and diversity 62%. The DARIS size was 3645 participants. The cumulative Z-curve (blue line) did not cross the trial sequential boundaries for benefit or harm nor the inner-wedge futility line (red outward sloping red lines) nor the DARIS. The light blue dotted lines show conventional boundaries (alpha 5%).

**Fig. S2.** Meta-analysis and TSA for proportion of participants without resolution of fluid overload for loop diuretics vs. placebo/no intervention

**
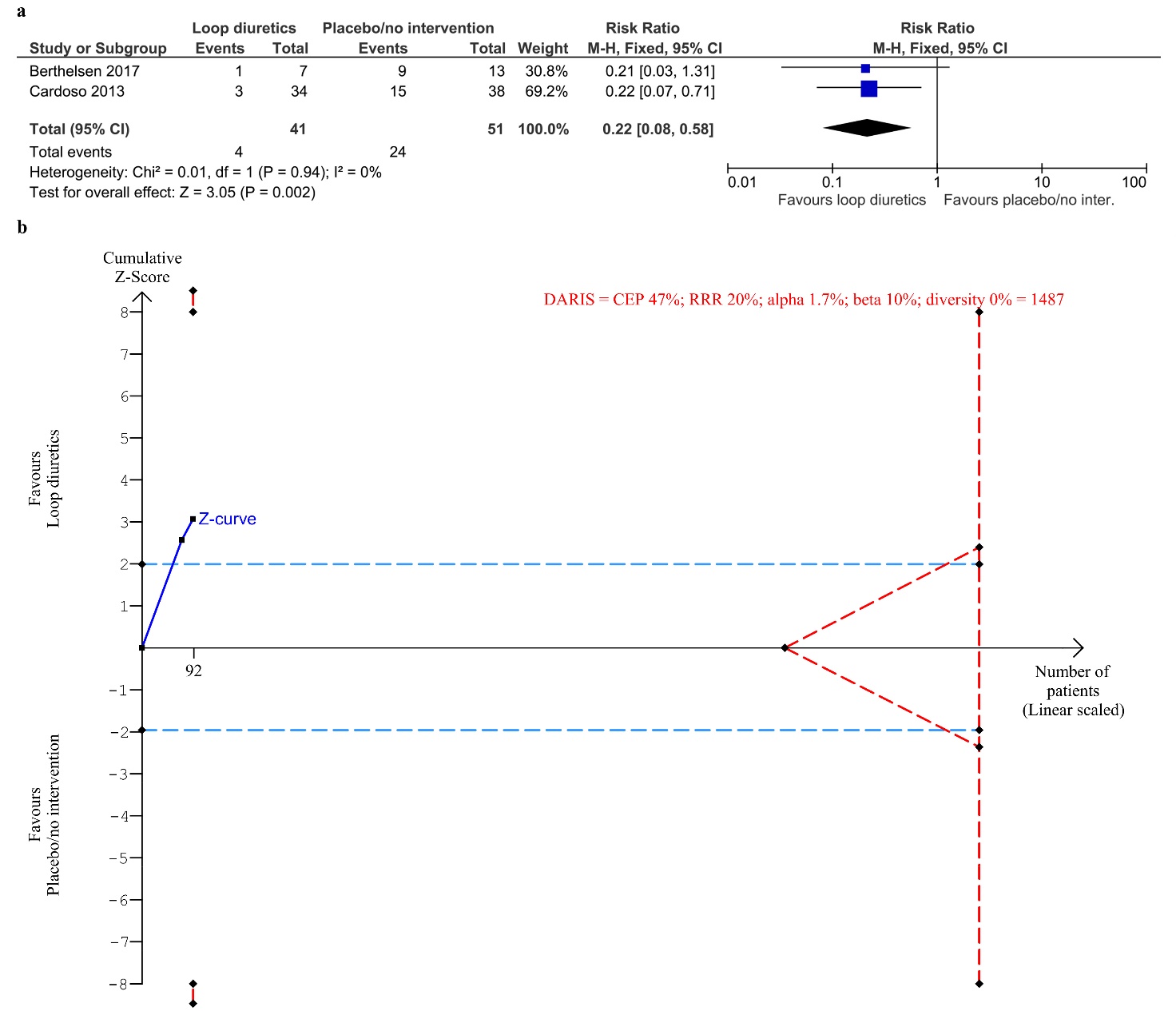
**

a) Meta-analysis. b) TSA. The diversity adjusted required information size (DARIS) was calculated according to the proportion of participants without resolution of fluid overload in the control group (CEP) of 47%; risk ratio reduction (RRR) of 20% in the experimental intervention group; alpha of 1.7%; a beta of 10% (90% power); and diversity 0%. The DARIS was 1487 participants. The cumulative Z-curve (blue line) did not cross the trial sequential boundaries for benefit or harm or the inner-wedge futility line (red outward sloping red lines) nor the DARIS. The light blue dotted lines show naïve conventional boundaries (alpha 5%).

**Fig. S3.** Meta-analysis for single serious adverse event - renal replacement therapy, for loop diuretics vs. placebo/no intervention

**
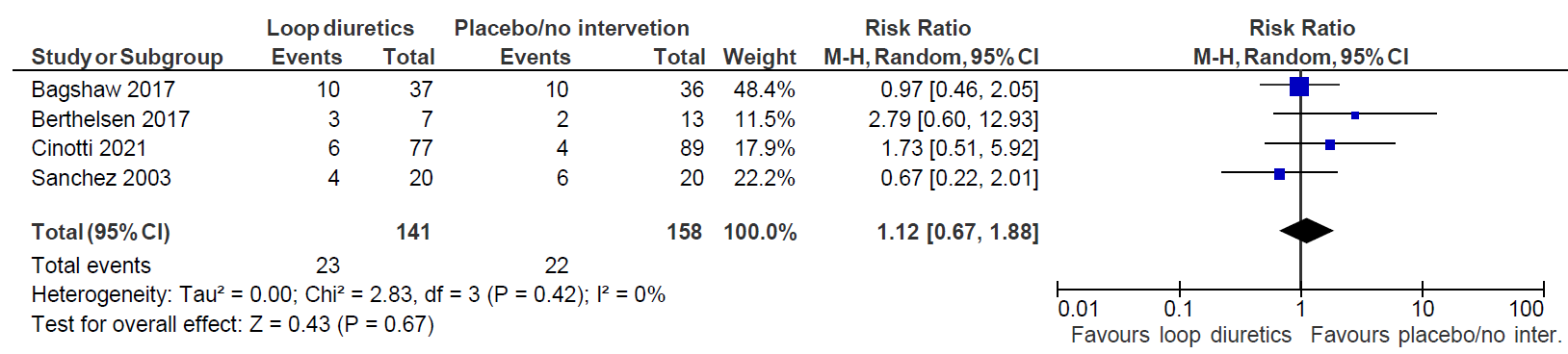
**

**Fig. S4.** Meta-analysis for single serious adverse event - worsening of acute kidney injury, for loop diuretics vs. placebo/no intervention

**
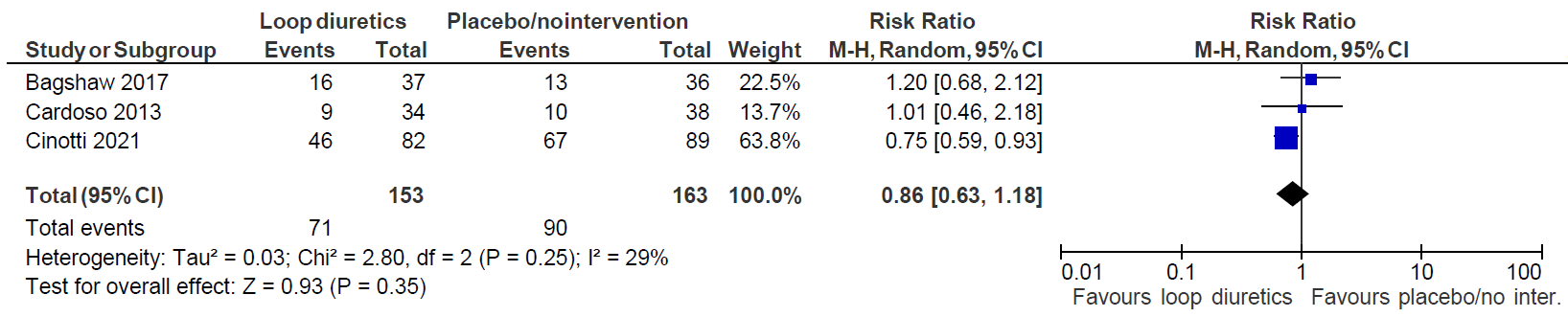
**

**Fig. S5.** Meta-analysis for single serious adverse event - atrial fibrillation, for loop diuretics vs. placebo/no intervention

**
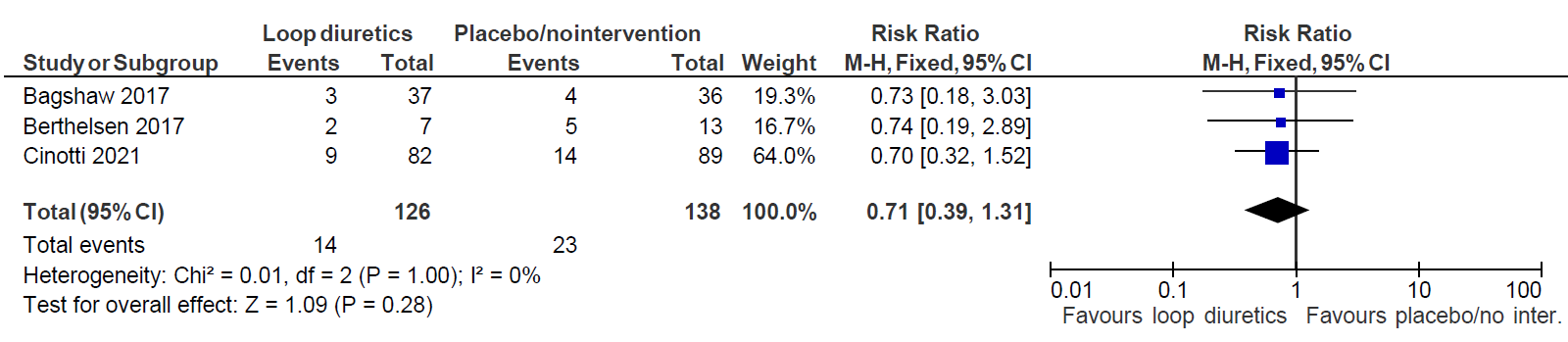
**

## **S6c. Subgroup analyses**

**Fig. S6.** Subgroup analysis of ICU diagnosis for all-cause mortality for loop diuretics vs. placebo/no intervention


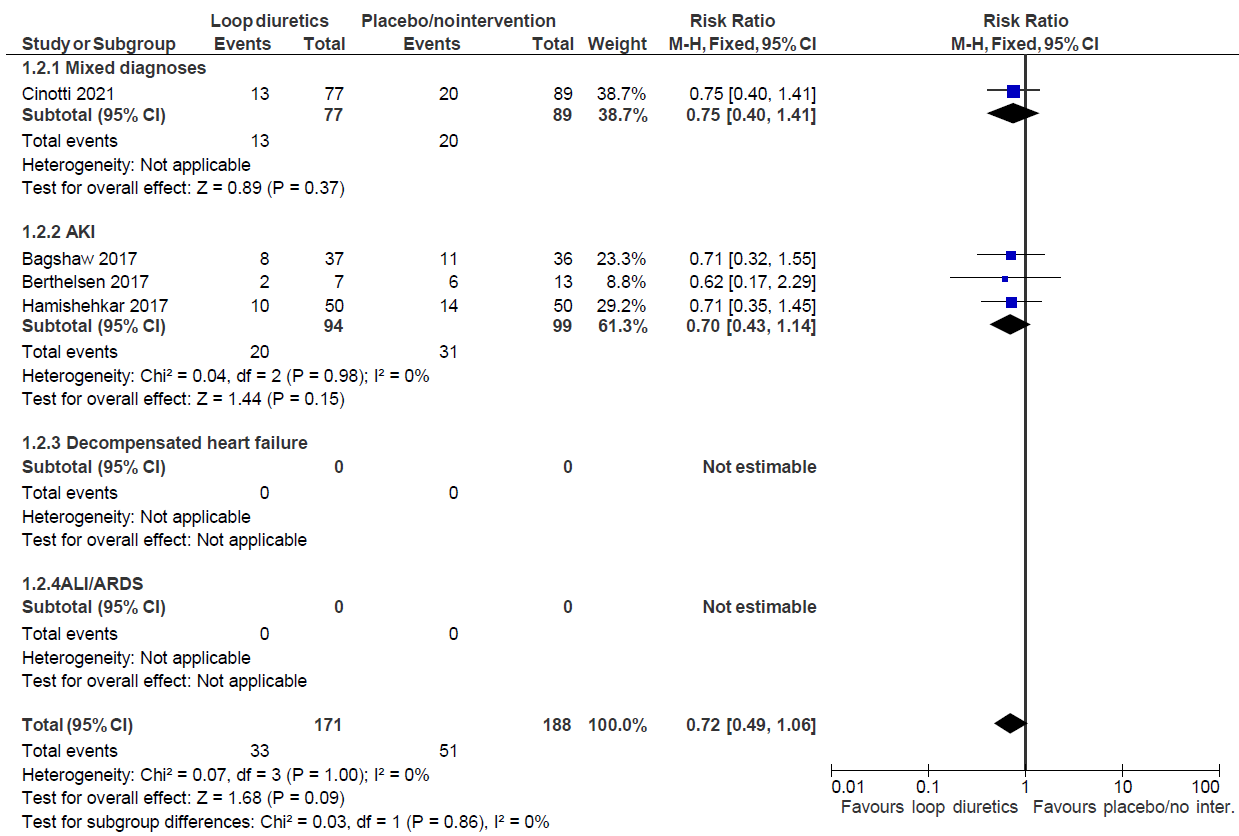


**Fig. S7.** Subgroup analysis of ICU population for all-cause mortality for loop diuretics vs. placebo/no intervention


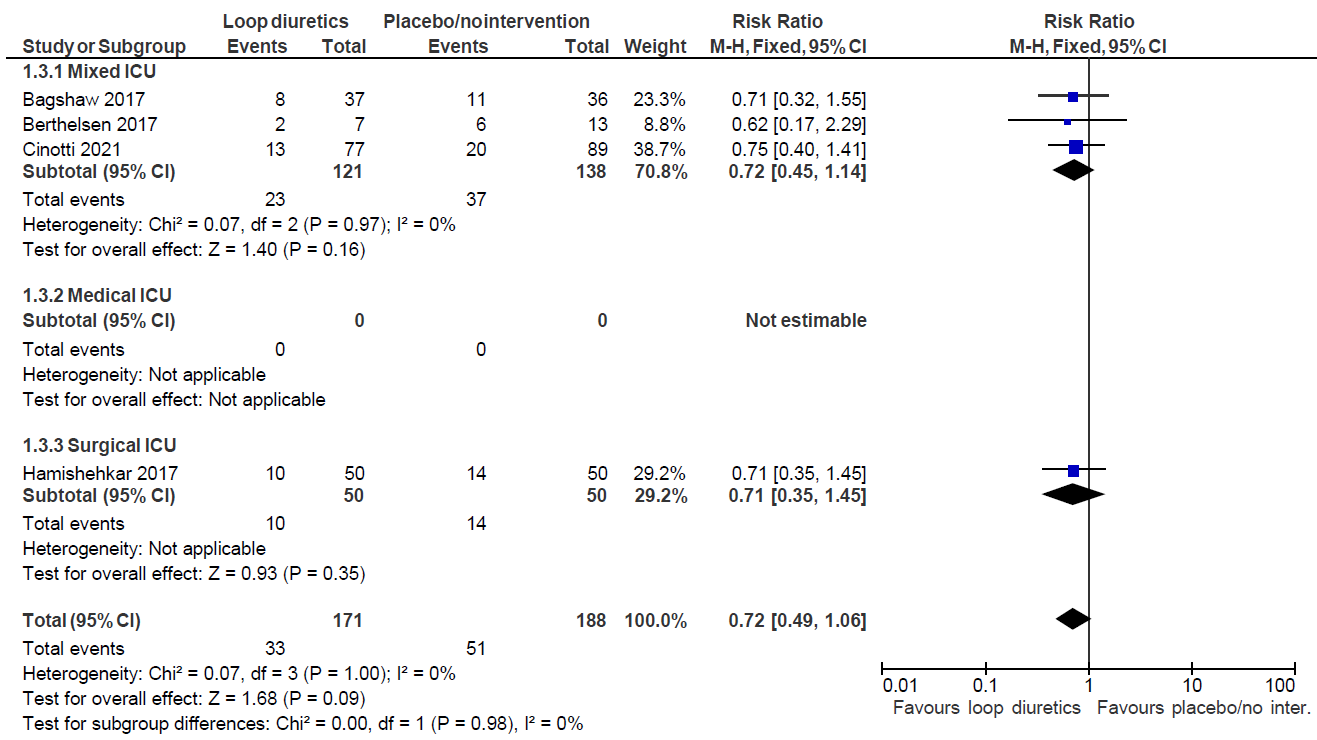


**Fig. S8** Subgroup analysis of severity of fluid overload for all-cause mortality for loop diuretics vs. placebo/no intervention


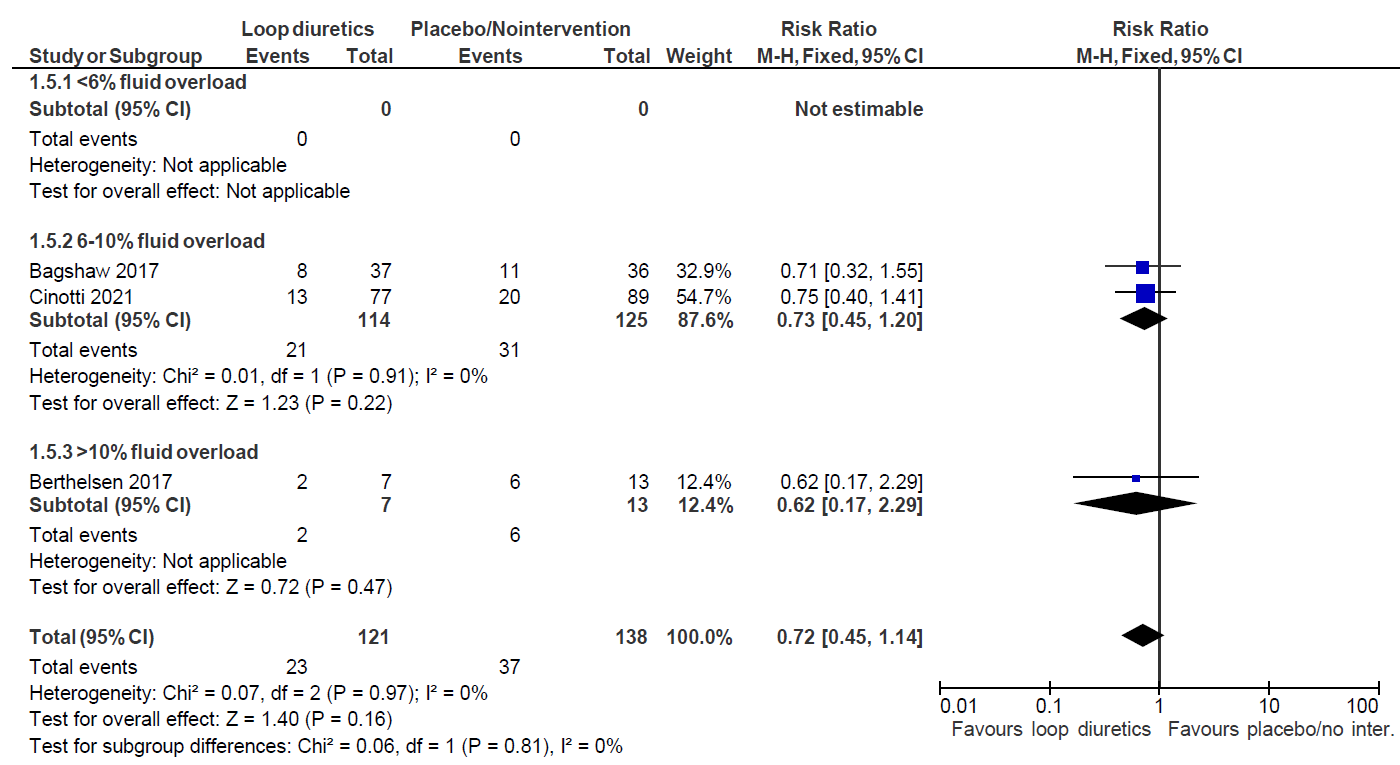


**Fig. S9.** Subgroup analysis of administration of diuretics in the control group for all-cause mortality for loop diuretics vs. placebo/no intervention

**
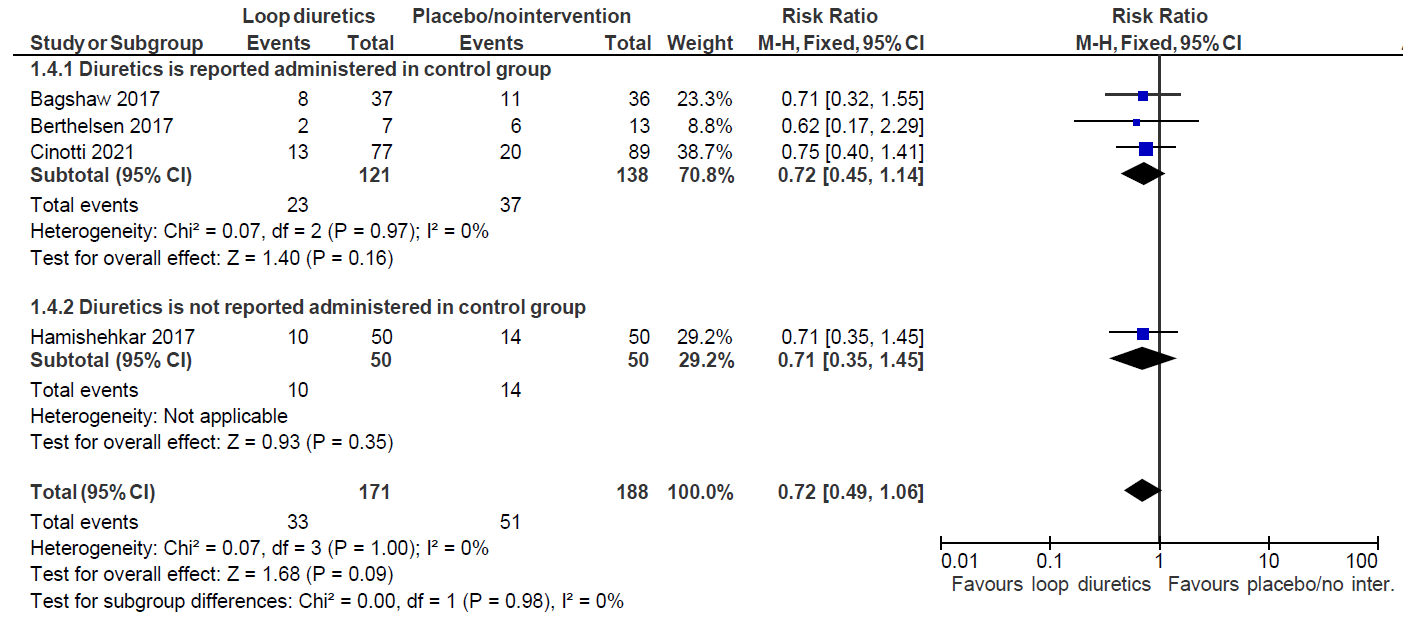
**

**Fig. S10.** Subgroup analysis of ICU diagnosis for serious adverse events for loop diuretics vs. placebo/no intervention


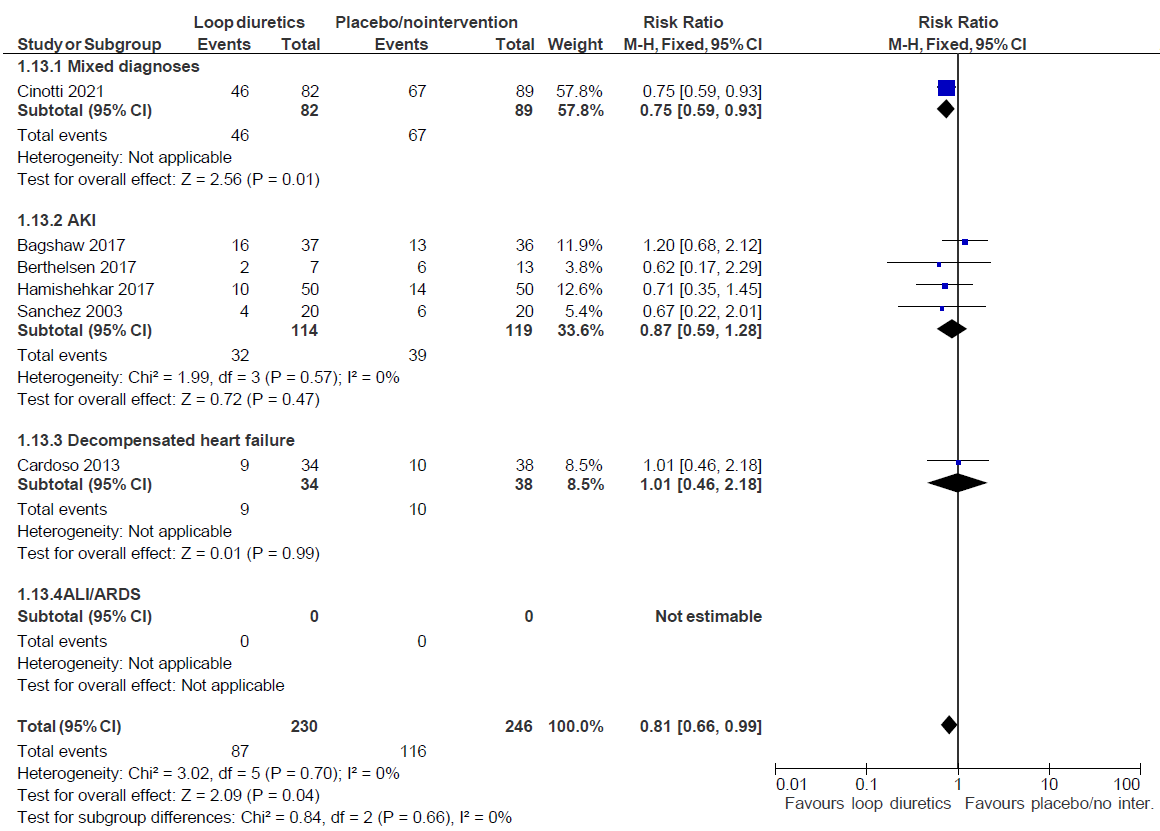


**Fig. S11.** Subgroup analysis of ICU population of serious adverse events for loop diuretics vs. placebo/no intervention

**
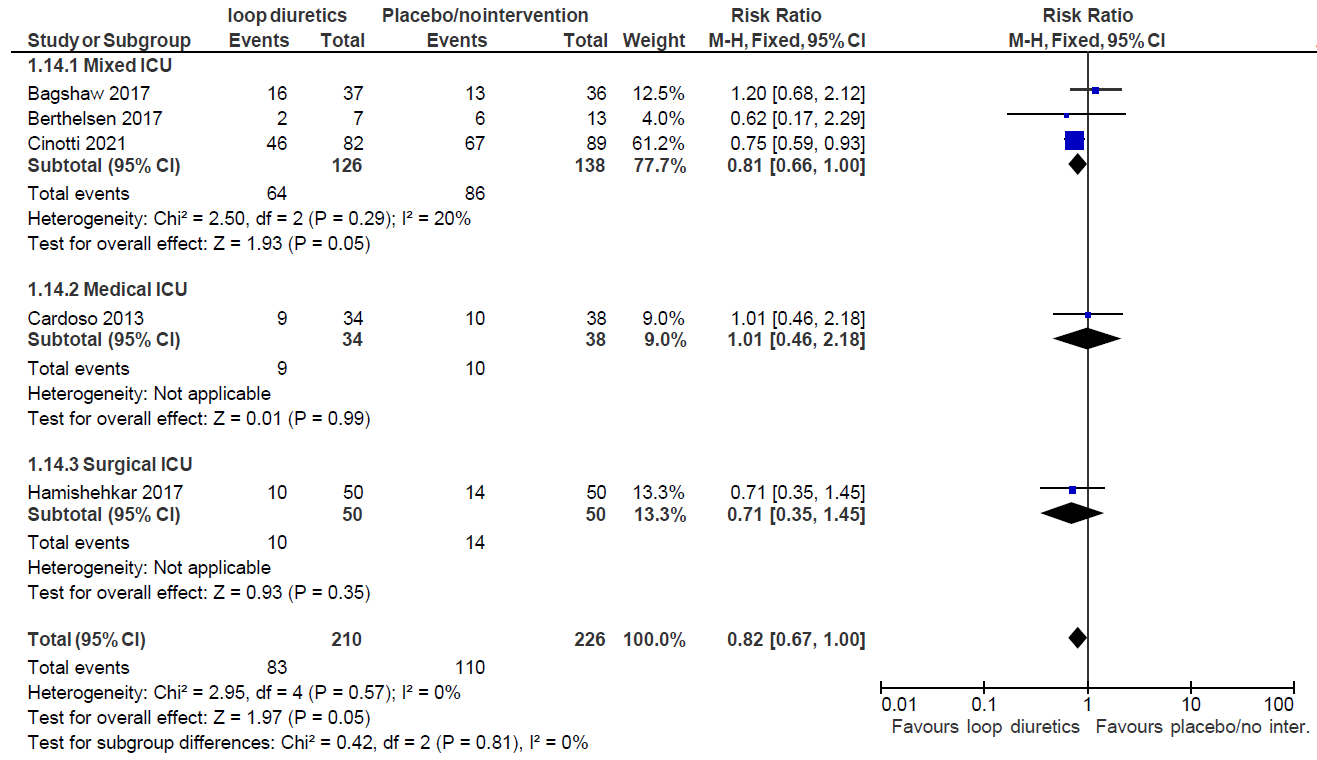
**

**Fig. S12. Subgroup analysis of severity of fluid overload for serious adverse events for loop diuretics vs. placebo/no intervention**

**
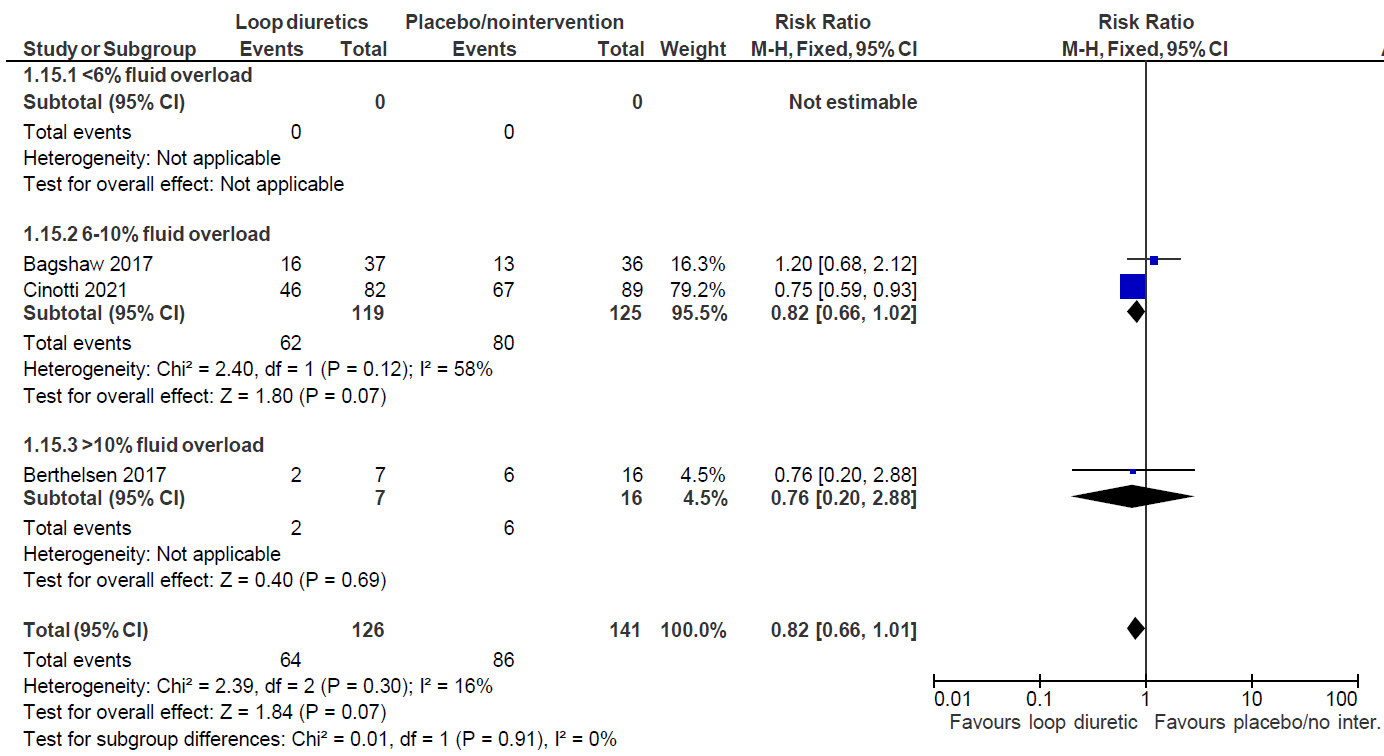
**

**Fig. S13.** Subgroup analysis of administration of diuretics in the control group for serious adverse events for loop diuretics vs. placebo/no intervention

**
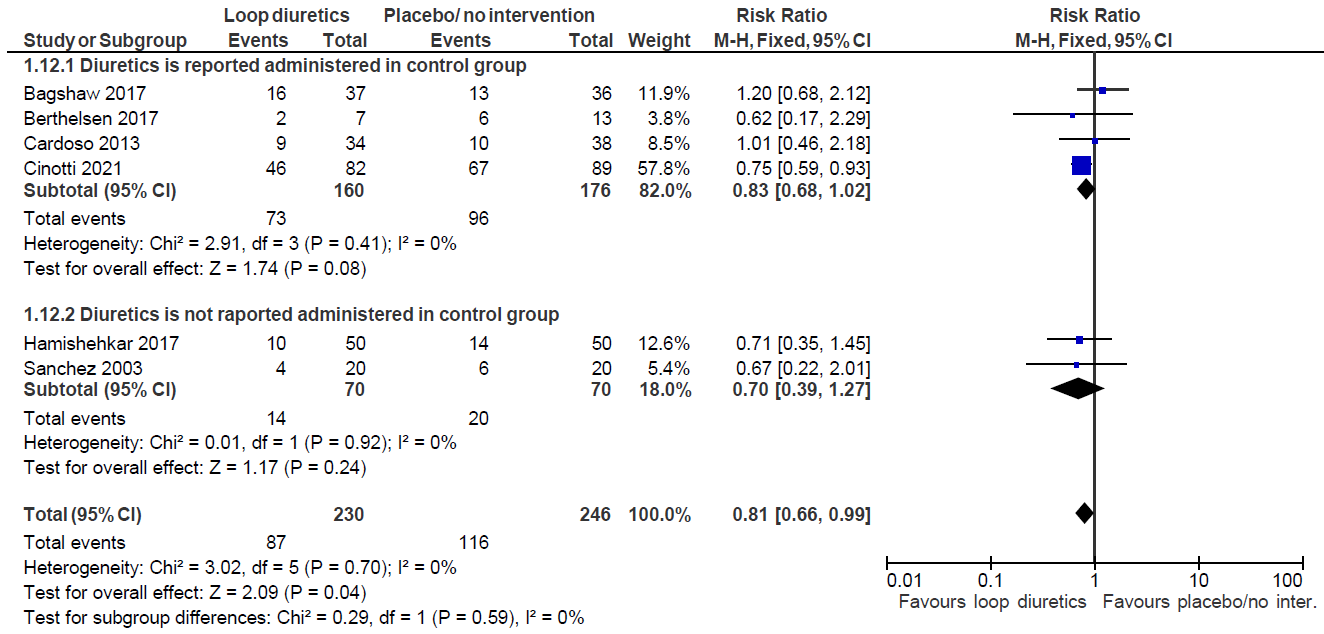
**

## **S6d. Sensitivity analyses**

**Fig. S14.** Sensitivity analysis, best – worst case scenario, of all-cause mortality for loop diuretics vs. placebo/no intervention

**
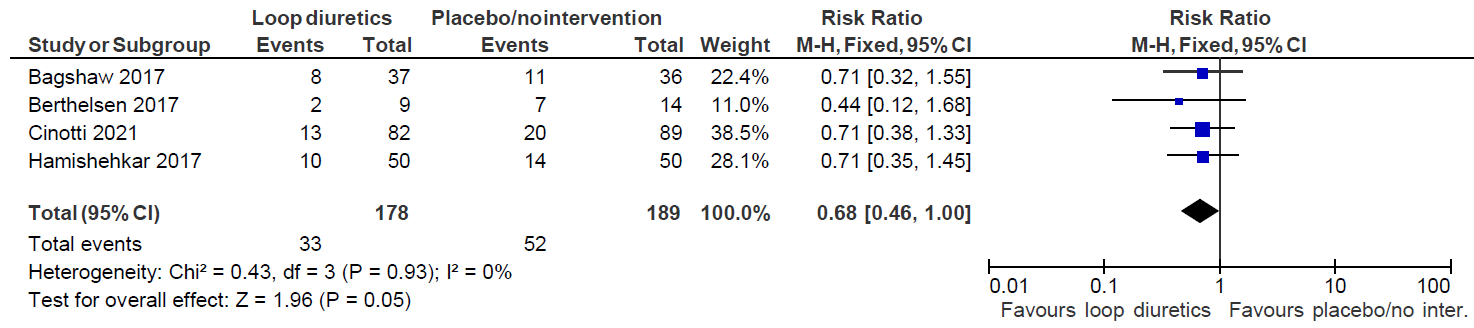
**

**Fig. S15.** Sensitivity analysis, worst – best case scenario, of all-cause mortality for loop diuretics vs. placebo/no intervention

**
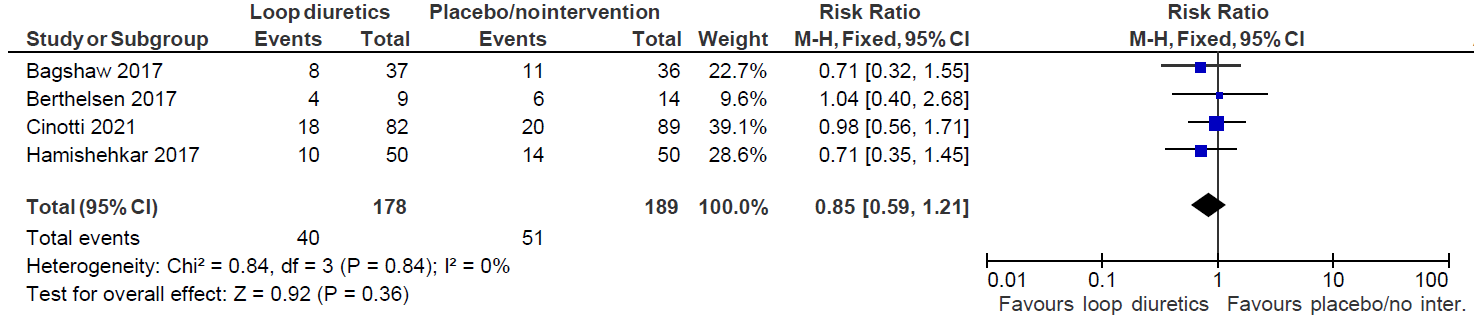
**

**Fig. S16.** Sensitivity analysis, best-worst case scenario, of serious adverse events for loop diuretics vs. placebo/no intervention

**
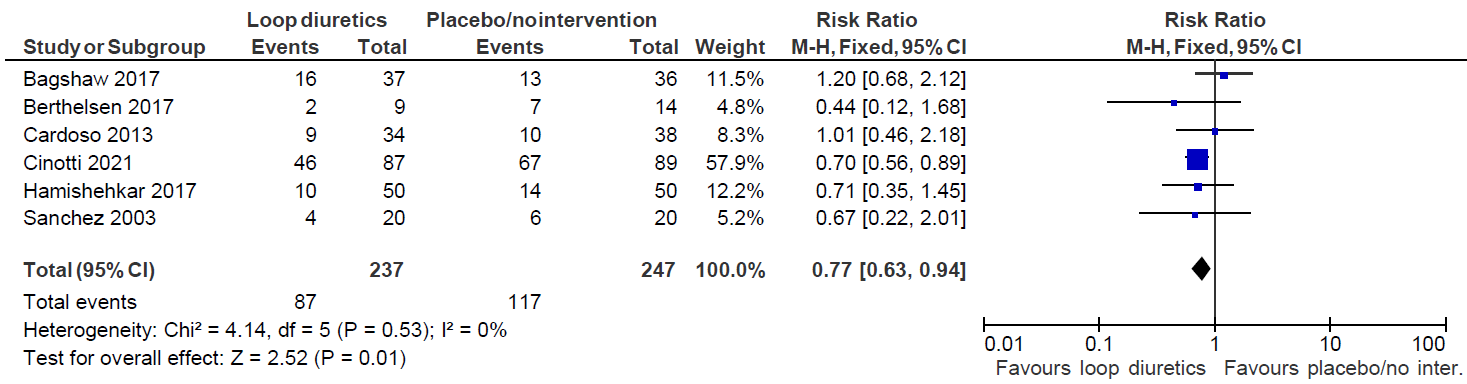
**

**Fig. S17.** Sensitivity analysis, worst - best case scenario, of serious adverse events for loop diuretics vs. placebo/no intervention

**
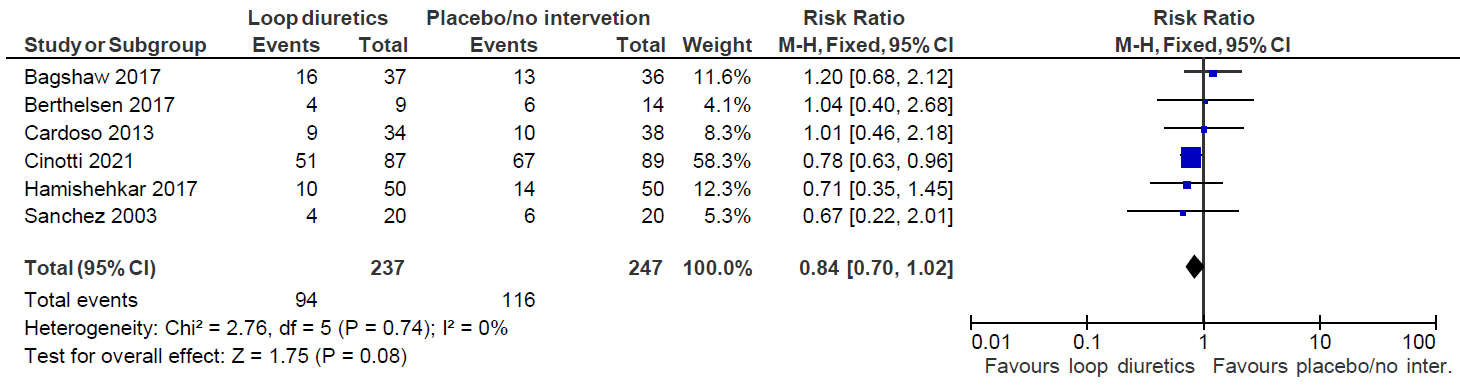
**

**Fig. S18.** Sensitivity analysis, best - worst scenario, of proportion of participants without resolution of fluid overload for loop diuretics vs. placebo/no intervention

**
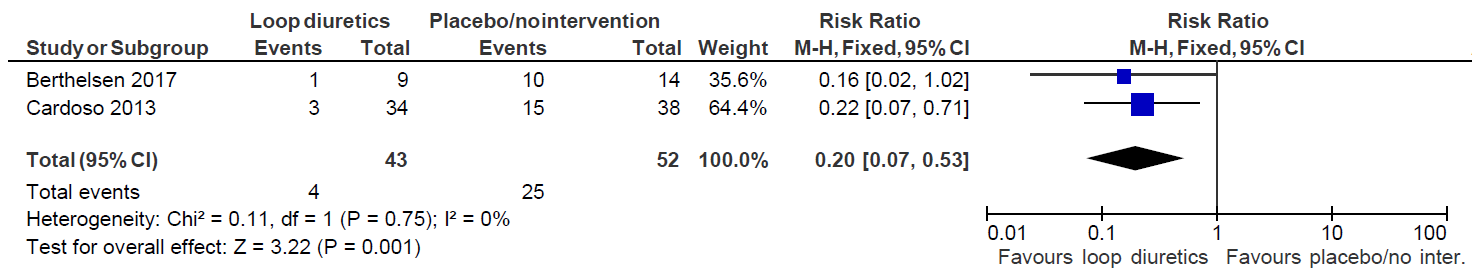
**

**Fig. S19.** Sensitivity analysis, worst - best scenario, of proportion of participants without resolution of fluid overload for loop diuretics vs. placebo/no intervention

**
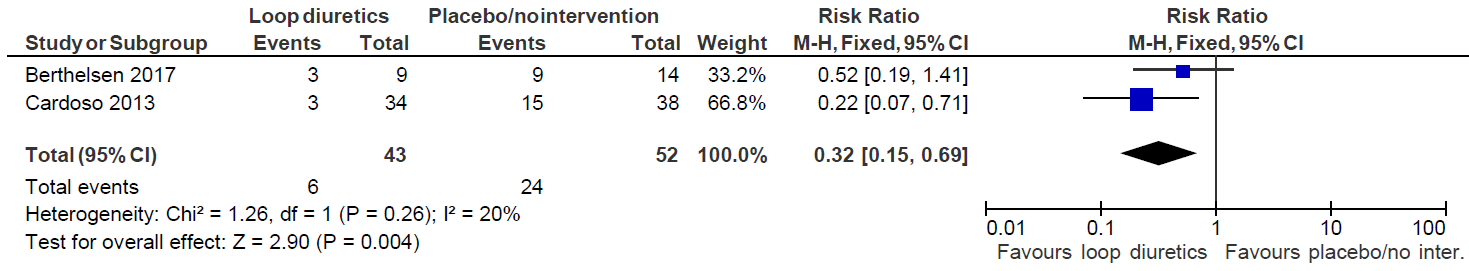
**

**Fig. S20.** Sensitivity analysis, best - worst scenario, of adverse events not considered serious for loop diuretics vs. placebo/no intervention

**
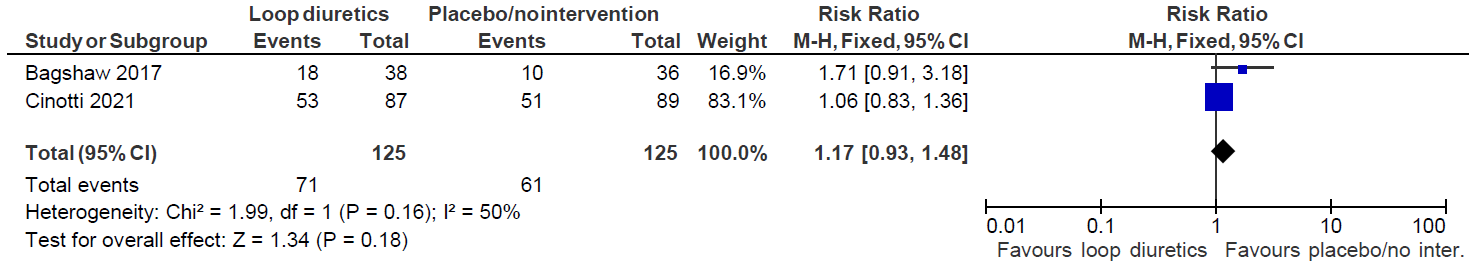
**

**Fig. S21.** Sensitivity analysis, worst - best scenario, of adverse events not considered serious for loop diuretics vs. placebo/no intervention

**
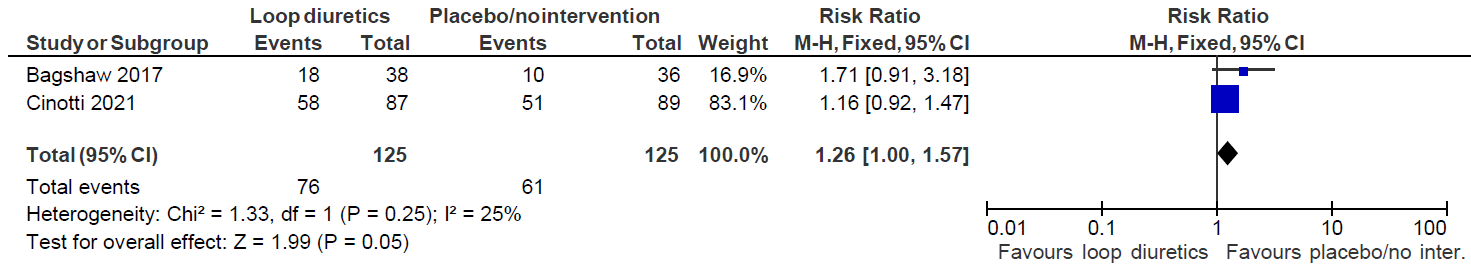
**

## **S6e. Reported SAEs and AEs**

Serious adverse events and adverse events not considered serious, which includes reactions to trial drug (serious adverse reaction and adverse reaction) are often not reported or not reported according to the ICH-GCP guidelines. If adverse events are reported, they are seldom divided in serious and not serious. We extracted all SAE and AE from the trials according to ICH-GCP guideline – also events not categorised as adverse events by the authors.

**Table S14.** Single SAEs and AEs for loop diuretics vs placebo/no intervention

| **Loop diuretics vs placebo/no intervention** | | | | |
| --- | --- | --- | --- | --- |
| **Trials** | **Single SAE** | **Intervention group** | **Control group** |  |
| Bagshaw [52] | RRT | 10 | 10 |  |
|  | Worsening of AKI | 16 | 13 |  |
|  | Ventricular Tachycardia/ventricular fibrillation | 2 | 0 |  |
|  | Tinnitus | 0 | 1 |  |
|  | Supraventricular tachycardia | 3 | 4 |  |
|  | Mortality | 8 | 11 |  |
| Berthelsen [37] | RRT | 3 | 2 |  |
|  | Atrial fibrillation | 2 | 5 |  |
|  | Ischaemic event | 1 | 1 |  |
|  | Anaemia requiring transfusion | 2 | 3 |  |
|  | Hypokalaemia | 2 | 0 |  |
|  | Thrombocytopenia < 50 x 10^9^/L | 2 | 2 |  |
|  | Pancreatitis | 0 | 1 |  |
|  | Seizure | 0 | 1 |  |
|  | Arrythmia | 1 | 3 |  |
|  | Mortality | 2 | 6 |  |
| Cardoso [55] | Worsening AKI | 9 | 10 |  |
| Cinotti [54] | RRT | 6 | 4 |  |
|  | Worsening of AKI | 46 | 67 |  |
|  | Atrial fibrillation | 9 | 14 |  |
|  | Torsade de pointes | 1 | 0 |  |
|  | Ventricular fibrillation | 1 | 2 |  |
|  | Ventricular tachycardia | 2 | 2 |  |
|  | Mortality | 13 | 20 |  |
| Hamishehkar [56] | Mortality | 10 | 14 |  |
| Sanchez [57] | RRT | 4 | 6 |  |
| **Trials** | **Single AE** | **Intervention group** | **Control group** |  |
| Bagshaw [52] | Drug reaction | 1 | 1 |  |
|  | Elevated lever enzymes | 0 | 1 |  |
|  | Serum sodium ≥ 150 mmol/L | 9 | 1 |  |
|  | Serum potassium < 3.0 mmol/L | 4 | 2 |  |
|  | Serum magnesium < 0.7 mmol/L | 2 | 4 |  |
|  | Serum bicarbonate ≥ 30 or pH ≥ 7.5 | 18 | 10 |  |
| Berthelsen [37] | - | - | - |  |
| Cardoso [55] | - | - | - |  |
| Cinotti [54] | Serum sodium ≥ 145 mmol/L | 40 | 40 |  |
|  | Serum sodium ≤135 mmol/L | 33 | 42 |  |
|  | Serum potassium ≤ 3.5 mmol/L | 53 | 51 |  |
| Hamishehkar [56] | - | - | - |  |
| Sanchez [57] | - | - | - |  |

Renal replacement therapy = RRT, Acute kidney injury = AKI

# **S7. Comparison: loop diuretics vs another loop diuretic**

## **S7a. Risk of bias**

**Table S15.** Risk of bias assessment on plasma creatinine for loop diuretics (furosemide) vs. another loop diuretic (piretanide or ethacrynic acid)

| **Study** | **Rando-misation** | **Deviations from intervention** | | **Missing outcome data** | **Measure-ment of the outcome** | **Selection of the reported results** | **Overall risk of bias** |
| --- | --- | --- | --- | --- | --- | --- | --- |
| Han | **Some concerns^1^** | | Low | Low | Low | **Some concerns^2^** | **High** |

1. Randomisation process not described.
2. Short protocol without statistical analysis plan was attached in the trial registry. One secondary outcome in the protocol was not reported in the article.

**Table S16.** Risk of bias assessment on plasma sodium and potassium for loop diuretics (furosemide) vs. another loop diuretic (piretanide or ethacrynic acid)

| **Study** | **Rando-misation** | **Deviations from intervention** | | **Missing outcome data** | **Measure-ment of the outcome** | **Selection of the reported results** | **Overall risk of bias** |
| --- | --- | --- | --- | --- | --- | --- | --- |
| Han | **Some concerns^1^** | | Low | Low | Low | **Some concerns^2^** | **High** |
| Wappler | **Some concerns^1^** | | **High^3^** | Low | Low | **Some concerns^4^** | **High** |

1. Randomisation process not described.
2. Short protocol without statistical analysis plan was attached in the trial registry. One secondary outcome in the protocol was not reported in the article.
3. No information about blinding or deviations for intended interventions.
4. No published protocol, statistical analysis plan or registration in a trial registry.

**Table S17.** Risk of bias assessment on serious adverse events for loop diuretics (furosemide) vs. another loop diuretic (piretanide or ethacrynic acid)

| **Study** | **Rando-misation** | **Deviations from intervention** | **Missing outcome data** | **Measure-ment of the outcome** | **Selection of the reported results** | **Overall risk of bias** |
| --- | --- | --- | --- | --- | --- | --- |
| Han | **Some concerns^1^** | Low | Low | Low | **Some concerns^2^** | **High** |

1. Randomisation process not described.
2. Short protocol without statistical analysis plan was attached in the trial registry. One secondary outcome in the protocol was not reported in the article.

**Table S18.** Risk of bias assessment on adverse events not considered serious for loop diuretics (furosemide) vs. another loop diuretic (piretanide or ethacrynic acid)

| **Study** | **Rando-misation** | **Deviations from intervention** | **Missing outcome data** | **Measure-ment of the outcome** | **Selection of the reported results** | **Overall risk of bias** |
| --- | --- | --- | --- | --- | --- | --- |
| Han | **Some concerns^1^** | Low | Low | Low | **Some concerns^2^** | **High** |

1. Randomisation process not described.
2. Short protocol without statistical analysis plan was attached in the trial registry. One secondary outcome in the protocol was not reported in the article.

## **S7b. Meta-analyses**

**Fig. S22.** Meta-analysis for plasma concentration of sodium for loop diuretics vs. another loop diuretic

**
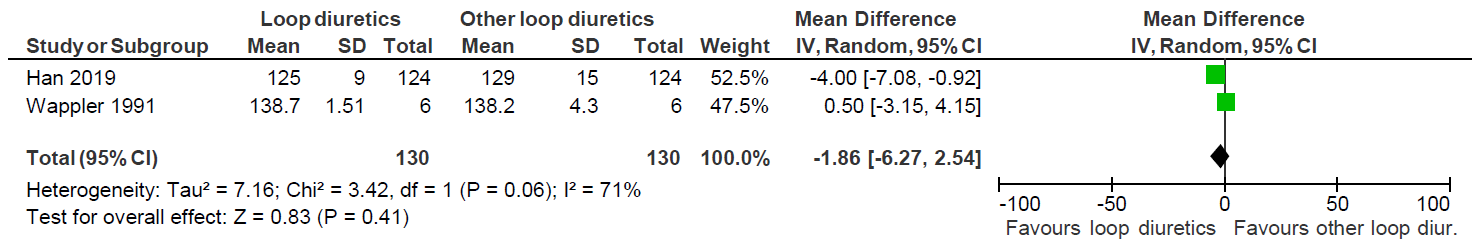
**

**Fig. S23.** Meta-analysis for plasma concentration of potassium for loop diuretics vs. another loop diuretic

**
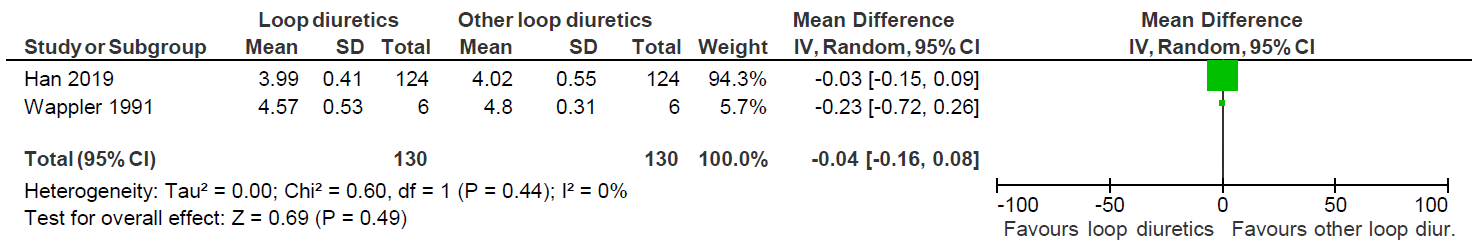
**

## **S7c. Narrative description of the results**

**Results for loop diuretics (furosemide) vs. another loop diuretic (piretanide or ethacrynic acid)**

Two trials compared loop diuretic vs another loop diuretic (260 participants) [58, 59]. Both trials represented patients from cardiac ICUs. One trial with 12 participants tested furosemide vs piretanide [58]. The other trial investigated furosemide vs ethacrynic acid in 248 participants [59]. The outcomes reported by the two trials were at overall high risk of bias. No data were reported on primary outcomes. Only two secondary outcomes (creatinine and AEs) were reported in one trial [59].

There was no difference in creatinine concentration between the group treated with loop diuretics vs another type of loop diuretic (ethacrynic acid).

One explorative outcome, concentration of sodium and potassium, were reported on in both trials. Meta-analysis showed no difference between the groups. No data was reported on serum chloride concentration.

## **S7d. Reported SAEs and AEs**

**Table S19.** Reported SAEs and AEs for loop diuretics vs. another diuretic

| **Loop diuretics vs another loop diuretic** | | | |
| --- | --- | --- | --- |
| **Trials** | **Single SAE** | **Intervention group** | **Control group** |
| Han [59] | Tinnitus and hearing loss | 15 | 23 |
|  | Seizure | 3 | 1 |
| Wappler [58] | - | - | - |
| **Trials** | **Single AE** | **Intervention group** | **Control group** |
| Han [59] | Hypocalcaemia | 8 | 0 |
|  | Hypomagnesemia | 9 | 0 |
|  | Oral and gastric irritation | 4 | 1 |
|  | Constipation | 4 | 1 |
|  | Blurred vision | 5 | 2 |
|  | Thrombophlebitis | 15 | 16 |
|  | Hypotension | 7 | 13 |
| Wappler [58] | - | - | - |

## **S7e. Summary of findings**

**Table S20.** Summary of findings for loop diuretics vs. another loop diuretic

| **Certainty assessment** | | | | | | | **No. of patients** | | **Effect** | | | **Certainty** | **Importance** |
| --- | --- | --- | --- | --- | --- | --- | --- | --- | --- | --- | --- | --- | --- |
| No. of studies | Study design | Risk of Bias | Inconsistency | Indirectness | Impreci-sion | Other considerations | Loop diuretics | Another loop diuretic | Relative (95% CI) | Absolute (95% CI) | |  |  |
| **All-cause mortality – not reported** | | | | | | | | | | | | | |
| - | - | - | - | - | - | - | - | - | - | - | | - | CRITICAL |
| **Quality of life – not reported** | | | | | | | | | | | | | |
| - | - | - | - | - | - | - | - | - | - | - | | - | CRITICAL |
| **Serious adverse events (SAE) – not reported** | | | | | | | | | | | | | |
| - | - | - | - | - | - | - | - | - | - | - | | - | CRITICAL |
| **Plasma concentration of creatinine** | | | | | | | | | | | | | |
| 1 | RCT | Serious^a^ | Not serious^b^ | Serious^c^ | Serious^d^ | None | 124 | 124 | No statistically significant difference in creatinine after 3 days | | | ⨁◯◯◯ VERY LOW | CRITICAL |
| **Proportion of participants without resolution of fluid overload – not reported** | | | | | | | | | | | | | |
| - | - | - | - | - | - | - | - | - | **-** | **-** | | - | CRITICAL |
| **Days in mechanical ventilation – not reported** | | | | | | | | | | | | | |
| - | - | - | - | - | - | - | - | - | **-** | - | | **-** | IMPORTANT |
| **Length of stay in ICU – not reported** | | | | | | | | | | | | | |
| - | - | - | - | - | - | - | - | - | - | - | | - | IMPORTANT |
| **Adverse event not considered serious (AE)** | | | | | | | | | | | | | |
| 1 | RCT | Serious^a^ | Not serious^b^ | Serious^c^ | Serious^d^ | None | 15/124 (12.1%) | 23/124 (18.5%) | - | | - | ⨁◯◯◯ VERY LOW | NOT IMPORTANT |

RCT: randomised clinical trials; CI: Confidence interval; RR: Risk ratio

a. The outcome was judged at overall high risk of bias

b. Cannot be assessed with only one trial.

c. This trial only included patients in cardiac ICU who were able to sign an informed consent. This leaves out other ICU populations and more severely ill patients not able to consent before entering the trial.

d. The total number of participants were 248 participants, which is concerning for imprecision.

# **S8. Comparison: loop diuretics vs. another type of diuretic**

## **S8a. Risk of bias**

**Table S21.** Risk of bias assessment on mortality for loop diuretics (furosemide) vs. another type of diuretic (acetazolamide or tolvaptan)

| **Study** | **Rando-misation** | **Deviations from intervention** | **Missing outcome data** | **Measure-ment of the outcome** | **Selection of the reported results** | **Overall risk of bias** |
| --- | --- | --- | --- | --- | --- | --- |
| Ng | **Some concerns^1^** | **High^2^** | **High^3^** | Low | **Some concerns^4^** | **High** |

1. Allocation process not described
2. No blinding. Only data on participants who remained on study protocol were registered. After the first 48 hours trial intervention was stopped in a large percentage of participants - mainly in the tolvaptan group due to clinical decisions (clinical resolution or diuretic switch to bumetanide) and additional medicine as metolazone was used more in the tolvaptan group compared to the furosemide group. This affected the outcomes with data from 48 hours to 96 hours. Per protocol analyses were performed.
3. No data on withdrawal or missing data. Mortality was not reported in the article.
4. Mortality was listed as an outcome in the protocol and article but only reported on ClinicalTrials.gov.

**Table S22.** Risk of bias assessment on serious adverse events for loop diuretics (furosemide) vs. another type of diuretic (acetazolamide or tolvaptan)

| **Study** | **Rando-misation** | **Deviations from intervention** | **Missing outcome data** | **Measure-ment of the outcome** | **Selection of the reported results** | **Overall risk of bias** |
| --- | --- | --- | --- | --- | --- | --- |
| Ng | **Some concerns^1^** | **High^2^** | Low | Low | **Some concerns^3^** | **High** |

1. Allocation process not described
2. No blinding. Only data on participants who remained on study protocol were registered. After the first 48 hours trial intervention was stopped in a large percentage of participants - mainly in the tolvaptan group due to clinical decisions (clinical resolution or diuretic switch to bumetanide) and additional medicine as metolazone was used more in the tolvaptan group compared to the furosemide group. This affected the outcomes with data from 48 hours to 96 hours. In the protocol it is stated that the collection of data continued in case of patients were taken out of the intervention due to safety. These data were not included in the analyses. Per protocol analyses were performed.
3. Three outcomes in the protocol were not reported in the article – some degree of selective reporting cannot be ruled out. SAE were only reported on ClincalTrials.gov – not in the article.

**Table S23.** Risk of bias assessment on plasma creatinine for loop diuretics (furosemide) vs. another type of diuretic (acetazolamide or tolvaptan)

| **Study** | **Rando-misation** | **Deviations from intervention** | **Missing outcome data** | **Measure-ment of the outcome** | **Selection of the reported results** | **Overall risk of bias** |
| --- | --- | --- | --- | --- | --- | --- |
| Ng | **Some concerns^1^** | **High^2^** | Low | Low | Low | **High** |

1. Allocation process not described
2. No blinding. Only data on participants who remained on study protocol were registered. After the first 48 hours trial intervention was stopped in a large percentage of participants - mainly in the tolvaptan group due to clinical decisions (clinical resolution or diuretic switch to bumetanide) and additional medicine as metolazone was used more in the tolvaptan group compared to the furosemide group. This affected the outcomes with data from 48 hours to 96 hours. Per protocol analyses were performed.

**Table S24.** Risk of bias assessment on adverse events not considered serious for loop diuretics (furosemide) vs. another type of diuretic (acetazolamide or tolvaptan)

| **Study** | **Rando-misation** | **Deviations from intervention** | **Missing outcome data** | **Measure-ment of the outcome** | **Selection of the reported results** | **Overall risk of bias** |
| --- | --- | --- | --- | --- | --- | --- |
| Ng | **Some concerns^1^** | **High^2^** | Low | Low | **Some concerns^3^** | **High** |

1. Allocation process not described
2. No blinding. Only data on participants who remained on study protocol were registered. After the first 48 hours trial intervention was stopped in a large percentage of participants - mainly in the tolvaptan group due to clinical decisions (clinical resolution or diuretic switch to bumetanide) and additional medicine as metolazone was used more in the tolvaptan group compared to the furosemide group. This affected the outcome data from 48 hours to 96 hours. Per protocol analyses were performed.
3. Three outcomes in the protocol were not reported in the article – some degree of selective reporting cannot be ruled out. AEs were not reported in the article but on ClincalTrials.gov.

**Table S25.** Risk of bias assessment on plasma sodium and potassium for loop diuretics (furosemide) vs. another type of diuretic (acetazolamide or tolvaptan)

| **Study** | **Rando-misation** | **Deviations from intervention** | **Missing outcome data** | **Measure-ment of the outcome** | **Selection of the reported results** | **Overall risk of bias** |
| --- | --- | --- | --- | --- | --- | --- |
| Brown | Low | Low | Low | Low | **Some concerns^1^** | **High** |
| Ng | **Some concerns^2^** | **High^3^** | Low | Low | Low | **High** |

1. No protocol or analysis plan is available, but the trial was registered at New Zealand Clinical Trials Registry. In the trial registry 15 secondary outcomes were listed but only 8 outcomes were reported in the article.
2. 1 Allocation process not described
3. No blinding. Only data on participants who remained on study protocol were registered. After the first 48 hours trial intervention was stopped in a large percentage of participants - mainly in the tolvaptan group due to clinical decisions (clinical resolution or diuretic switch to bumetanide) and additional medicine as metolazone was used more in the tolvaptan group compared to the furosemide group. This affected the outcome data from 48 hours to 96 hours. Per protocol analyses were performed.

**Table S26.** Risk of bias assessment on plasma chloride for loop diuretics (furosemide) vs. another type of diuretic (acetazolamide or tolvaptan)

| **Study** | **Rando-misation** | **Deviations from intervention** | **Missing outcome data** | **Measure-ment of the outcome** | **Selection of the reported results** | **Overall risk of bias** |
| --- | --- | --- | --- | --- | --- | --- |
| Brown | Low | Low | Low | Low | **Some concerns^1^** | **High** |

1. No protocol or analysis plan is available, but the trial was registered at New Zealand Clinical Trials Registry. In the trial registry 15 secondary outcomes were listed but only 8 outcomes were reported in the article.

## **S8b. Narrative description of the results**

**Results for loop diuretic (furosemide) vs. another type of diuretic (acetazolamide or tolvaptan)**

Two trials compared loop diuretics with another type of diuretic (58 participants) [53, 60]. One trial included mixed ICU patients and investigated the effects of furosemide vs acetazolamide over a study time of just 6 hours [53]. The other trial included patients with decompensated hearth failure in a medical ICU investigating furosemide vs tolvaptan for up to 96 hours [60]. All outcomes for both trials were at overall high risk of bias. No meta-analyses could be performed on any outcomes.

The trial testing tolvaptan [60] found a mortality of 0% in both groups. No data reported on the remaining primary outcomes. The only secondary outcome reported was plasma concentration of creatinine and of explorative outcomes: plasma concentration of sodium, potassium and single AE. No difference between the group treated with furosemide vs tolvaptan was found for plasma concentration of creatinine and potassium, but plasma sodium concentration decreased statistically significant in the group treated with furosemide and increased in the tolvaptan group. One AE was reported. All outcomes were at overall high risk of bias.

The trial testing acetazolamide [53] did not report on any of our primary outcomes. They only reported on plasma creatinine, sodium, potassium, and chloride concentrations. The found no difference between the group treated with loop diuretics vs acetazolamide [53]. All outcomes were at overall high risk of bias.

## **S8c. Reported SAEs and AEs**

**Table S27.** SAEs and AEs for loop diuretics vs. another type of diuretic

| **Loop diuretics vs another type of diuretic** | | | | | |
| --- | --- | --- | --- | --- | --- |
| **Trials** | **Single SAE** | **Intervention group** | **Control group** | | |
| Brown [53] | - | - | | | - |
| Ng [60] | Mortality | 0 | | | 0 |
| **Trials** | **Single AE** | **Intervention group** | **Control group** | | |
| Brown [53] | - | - | | - | |
| Ng [60] | Plasma creatinine increase > 26.5 mmol/L | 5 | | 3 | |

## **S8d. Summary of findings**

**Table S28.** Summary of findings for loop diuretics vs another type of diuretic

| **Certainty assessment** | | | | | | | **No. of patients** | | **Effect** | | **Certainty** | **Importance** |
| --- | --- | --- | --- | --- | --- | --- | --- | --- | --- | --- | --- | --- |
| No. of studies | Study design | Risk of Bias | Inconsistency | Indirect-ness | Imprecision | Other considera-tions | Loop diuretics | Another type of diuretic | Relative (95% CI) | Absolute (95% CI) |  |  |
| **All-cause mortality – not reported** | | | | | | | | | | | | |
| 1 | RCT | Very serious^a^ | Not serious^b^ | Serious^c^ | Very serious^d^ | None | 0/15 (0.0%) | 0/18 (0.0%) | - | - | - | CRITICAL |
| **Quality of life – not reported** | | | | | | | | | | | | |
| - | - | - | - | - | - | - | - | - | - | - | - | CRITICAL |
| **Serious adverse events (SAE) – not reported** | | | | | | | | | | | | |
| 1^e^ | RCT | Very serious^a^ | Not serious^b^ | Serious^c^ | Very serious^d^ | None | 0/15 (0.0%) | 0/18 (0.0%) | - | - | - | CRITICAL |
| **Plasma concentration of creatinine** | | | | | | | | | | | | |
| 2^f^ | RCT | Serious^g^ | Not serious | Serious^h^ | Very serious^i^ | None | - | - | The trials found no significant difference in plasma creatinine at longest follow up. | | ⨁◯◯◯ VERY LOW | CRITICAL |
| **Proportion of participants without resolution of fluid overload – not reported** | | | | | | | | | | | | |
| - | - | - | - | - | - | - | - | - | **-** | **-** | - | CRITICAL |
| **Days in mechanical ventilation – not reported** | | | | | | | | | | | | |
| - | - | - | - | - | - | - | - | - | **-** | - | **-** | IMPORTANT |
| **Length of stay in ICU – not reported** | | | | | | | | | | | | |
| - | - | - | - | - | - | - | - | - | - | - | - | IMPORTANT |
| **Adverse event not considered serious (AE)** | | | | | | | | | | | | |
| 1 | RCT | Serious^j^ | Not serious^b^ | Serious^c^ | Very serious^d^ | None | 5/15 (33.3%) | 3/18 (16.7%) | The trial showed no significant difference in the highest event rate of a single AE between groups. | | ⨁◯◯◯ VERY LOW | NOT IMPORTANT |

RCT: randomised clinical trials; CI: Confidence interval; RR: Risk ratio

a. The trial was at overall high risk of bias for this outcome. Mortality was listed as an outcome but not reported in the article only registered on ClinicalTrials.gov.

b. Cannot be assessed with only one trial

c. This trial consists of a subgroup of ICU patients with congestive heart failure able sign a consent form before enrollment. This is not representative for the ICU population in general.

d. The total number of participants were 33 participants, which is concerning for imprecision.

e. The review group registered mortality as an SAE. The mortality was reported as 0% in both groups on ClinicalTrials.gov.

f. A meta-analysis could not be performed because of unsuitable data.

g. All trials were at overall high risk of bias for this outcome

h. One trial lasted 6 hours and the other trial up to 96 hours. The time frame and different exposure to diuretics in dose and type is the reason for our judgment as serious inconsistency.

i. The total number of participants were 58 participants, which is concerning for impression.

j. The trial was at overall high risk of bias for this outcome
